# *In silico* analysis of potential off-target sites to gene editing for Mucopolysaccharidosis type I using the CRISPR/Cas9 system: Implications for population-specific treatments

**DOI:** 10.1371/journal.pone.0262299

**Published:** 2022-01-24

**Authors:** Paola Carneiro, Martiela Vaz de Freitas, Ursula Matte

**Affiliations:** 1 Post-Graduation Program on Genetics and Molecular Biology, Federal University of Rio Grande do Sul, Porto Alegre, Rio Grande do Sul, Brazil; 2 Bioinformatics Core, Experimental Research Center, Hospital de Clínicas, Porto Alegre, Rio Grande do Sul, Brazil; 3 Cell, Tissue and Genes Laboratory, Experimental Research Center, Hospital de Clínicas de Porto Alegre, Porto Alegre, Rio Grande do Sul, Brazil; 4 Department of Genetics, Federal University of Rio Grande do Sul, Porto Alegre, Rio Grande do Sul, Brazil; New England Biolabs Inc, UNITED STATES

## Abstract

Mucopolysaccharidosis type I (MPS I) is caused by alpha-L-iduronidase deficiency encoded by the *IDUA* gene. Therapy with CRISPR/Cas9 is being developed for treatment, however a detailed investigation of off-target effects must be performed. This study aims to evaluate possible off-targets for a sgRNA aiming to correct the most common variant found in MPS I patients (p.Trp402*). A total of 272 potential off-target sequences was obtained and 84 polymorphic sites were identified in these sequences with a frequency equal to or greater than 1% in at least one of the populations. In the majority of cases, polymorphic sites decrease the chance of off-target cleavage and a new PAM was created, which indicates the importance of such analysis. This study highlights the importance of screening off-targets in a population-specific context using Mucopolysaccharidosis type I as an example of a problem that concerns all therapeutic treatments. Our results can have broader applications for other targets already clinically in use, as they could affect CRISPR/Cas9 safety and efficiency.

## Introduction

Mucopolysaccharidosis type I (MPS I) is an autosomal recessive disease caused by the deficiency of alpha-L-iduronidase (IDUA; EC 3.2.1.76), an enzyme coded by the *IDUA* gene. The intra lysosomal accumulation of heparan and dermatan sulfate affects cellular homeostasis and triggers a multisystem dysfunction [1]. Three syndromes are associated with the clinical spectrum from severe to attenuate: Hurler (OMIM #67014), Hurler-Scheie (OMIM #607015), Scheie (OMIM # 67016). Several pathogenic mutations were identified in the *IDUA* gene and p.Trp402* is the most common variant in MPS I patients in Western countries [2–4]. This null allele results in the absence of the gene product [5] and is present in all three disease forms. Therefore, it is an interesting target for gene editing [6]. Therapy with Clustered Regularly Interspaced Short Palindromic Repeats associated with Cas9 proteins (CRISPR/Cas9) is under development to treat MPS I, with promising results both in human cells [7] and in an animal model of the disease [8].

Genome editing technologies have grown as an alternative to treat monogenic disorders. Among these, CRISPR/Cas9 won the field’s preference when compared with Transcription Activator-like Effector Nucleases (TALEN) and ZINC Finger. This preference relies on the more straightforward and less expensive construction, with no need for structural validation [9]. CRISPR/Cas9 is derived from prokaryotes’ immune mechanism and promotes the cleavage of specific regions adjacent upstream or downstream to a protospacer adjacent motif sequence (PAM) NGG for *Streptococcus pyogenes* guided by RNA (sgRNA) that contains the 20 nt complementary to the on-target site. Several features influence cleavage efficiency: GC content, having a G in the 20^th^ position in the sgRNA, number and position of mismatches, and the presence of PAM [10]. The number of mismatches is essential, and Tsai *et al*. showed that sequences with up to 3 mismatches are more likely to be cut during the editing [11]. Conservation of the first nucleotides adjacent to PAM (SEED) is also an important aspect [12, 13]. There is no consensus about the SEED’s length; however, some experimental assays showed that sequences with the conservation of the first five nucleotides adjacent to PAM could be cleaved [13]. Nevertheless, the possibility that other similar sites could be cleaved imposes a detailed investigation on these potential off-targets that must be performed before CRISPR/Cas9 reaches clinical application [14].

Many *in silico* tools were developed to investigate possible off-target regions during the on-target site editing using CRISPR/Cas9. However, results differ among them due to the different algorithms and criteria used by the predictors. Moreover, there is a need to consider the variability among the individuals in search for off-target sites. It has been shown that genetic variability can affect the cleavage process in the on-target region and similar sites [15, 16]. It is, therefore, likely that this variability can interfere with the prediction provided by the software.

This work aims to evaluate possible off-target regions for a sgRNA designed to correct the most common variant found in MPS I patients using the five most used *in silico* predictors for the CRISPR/Cas9 system [17]. Besides, we analyzed how polymorphic variants in different populations may affect the predicted off-target sites.

## Material and methods

**Fig 1:**
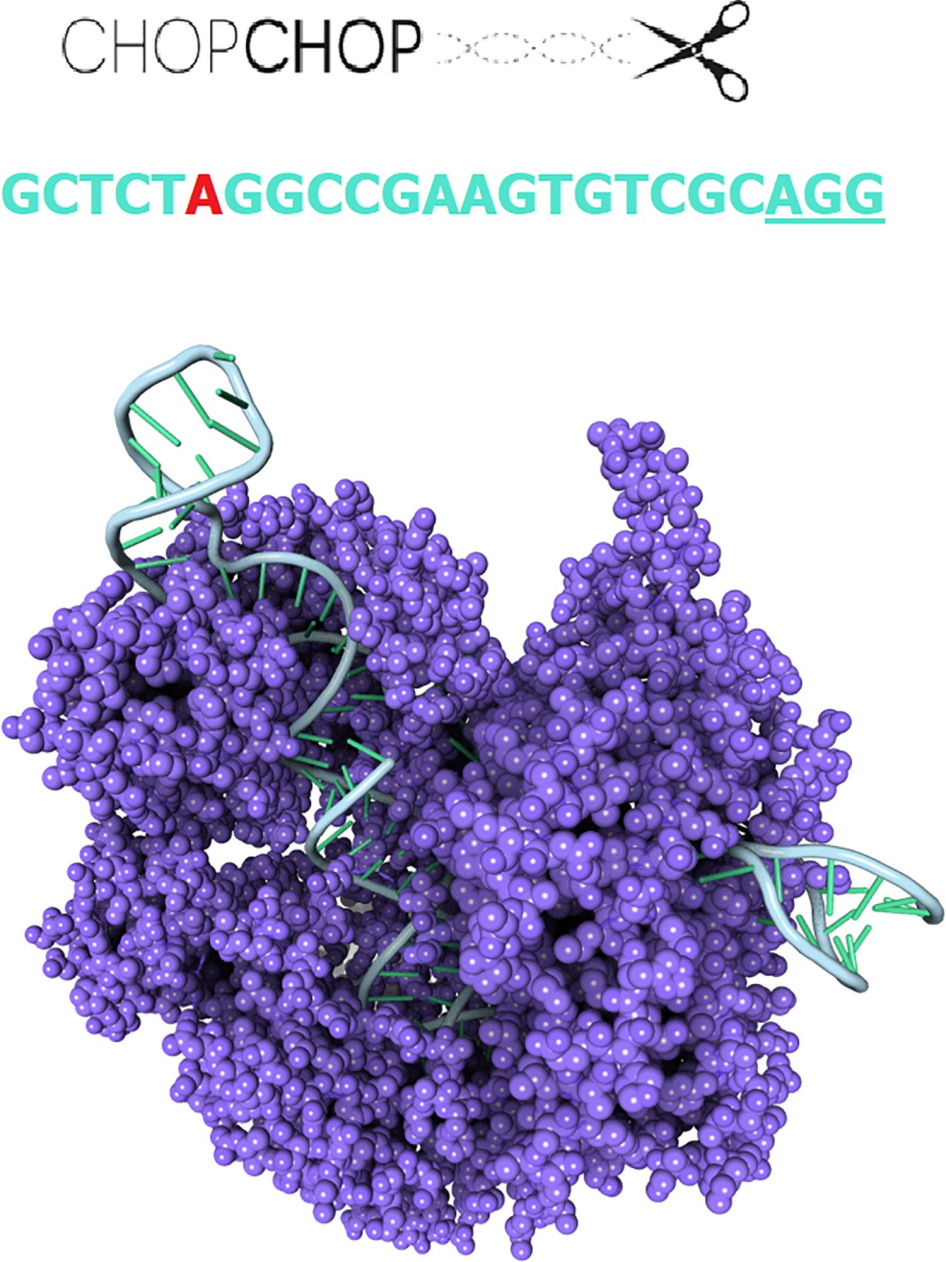
Schematic illustration of CRISPR/Cas9 system and the design of sgRNA with PAM sequence. The illustration shows the system’s components sgRNA linked to DNA (blue), PAM sequence (underline) and Cas9 (purple) (PDB ID 4OO8) [19], with a detailed view of the sgRNA above, with target variant p.Trp402*(red) and PAM sequence (underlined at the end of the sequence). Illustration of system components made using 3D Protein Imaging [20].

The sgRNA for the target region was designed using CHOPCHOP [18], and is located inside the *IDUA* gene (996530–996552, Ghc37/hg19). The sgRNA sequence is 20 nucleotides long and 65% GC content (Fig 1). A full description can be found in De Carvalho et al. [7].

The five most employed *in silico* tools for off-target prediction, according to Freitas *et al*. [17], were used: CHOPCHOP [18], COSMID [21], Cas-OFFinder [22], CCTop [23] and CRISPOR [24]. The choice of potential off-target sequences was based on the software’s score or the conservation of 5 nucleotides upstream PAM. We defined that off-target sequences could have up to 6 mismatches and up to 2 indels, following GUIDE-seq experimental evidence of cleaved sequences with 6 mismatches [11] and up to 2 insertions [25].

Then, sequences were aligned with the human genome Ghc37/hg19 version to confirm the region localization, as some tools only return the cutting position. After, the sequences with mismatches only were submitted to the CFD score [26] to evaluate the probability of that off-target being cut during gene editing. To evaluate this same probability for sequences with mismatches and indels, we used the CFD indel score, which was created by us and is available as supplementary files, based on Doench et al.’s results to calculate indels and CFD score for mismatches. Then, we proceed with the CFD final score to evaluate the cleavage probability by multiplying the probabilities of each event. The evaluation of the CFD final score considers the Doench et. al’s threshold (0.2) and that of Haeussler et al. (0.023) as probability of cleavage.

Then, the presence of polymorphic sites within the possible off-target sequences was evaluated for three populations (Europe, Africa, and Latin America) and the worldwide population. This evaluation was made using the population database 1000 Genomes phase 3 with the hg19 genomic version [27]. Until March 2020, the platform contained 2,504 individuals from different populations and 5,008 available alleles not related to sex chromosomes. To sexual chromosomes, the database contains 3,775 and 1,233 alleles provided for X and Y evaluation, respectively. An additional evaluation of variants present in Latin America was made using ABraOM–SABE609 [28] and LOVD version 3.0—Argentina DNA Variant Database with the Ghc37/hg19 genomic version. Only variants with allele frequencies equal to or greater than 1% in at least one of the three populations were considered polymorphic sites.

To investigate if polymorphic sites would create new PAM sequences, the sgRNA was aligned with the human genome by Burrows-Wheeler Aligner (BWA) version 0.7.17 [29]. For each possible PAM (NNG or NGN), the presence of a polymorphic site creating a new PAM (NGG) was searched on the databases mentioned above. For this evaluation, we considered only off-target sequences with six mismatches without indels and conserved SEED regions.

The evaluation of repetitive elements in the off-target sequences was made using RepeatMasker version 4.0.7 in the command line interface by DFAM [30] and Repbase [31] databases. The off-target sequences were downloaded from NCBI with 200 bp upstream and downstream flanking regions.

## Results

**Fig 2:**
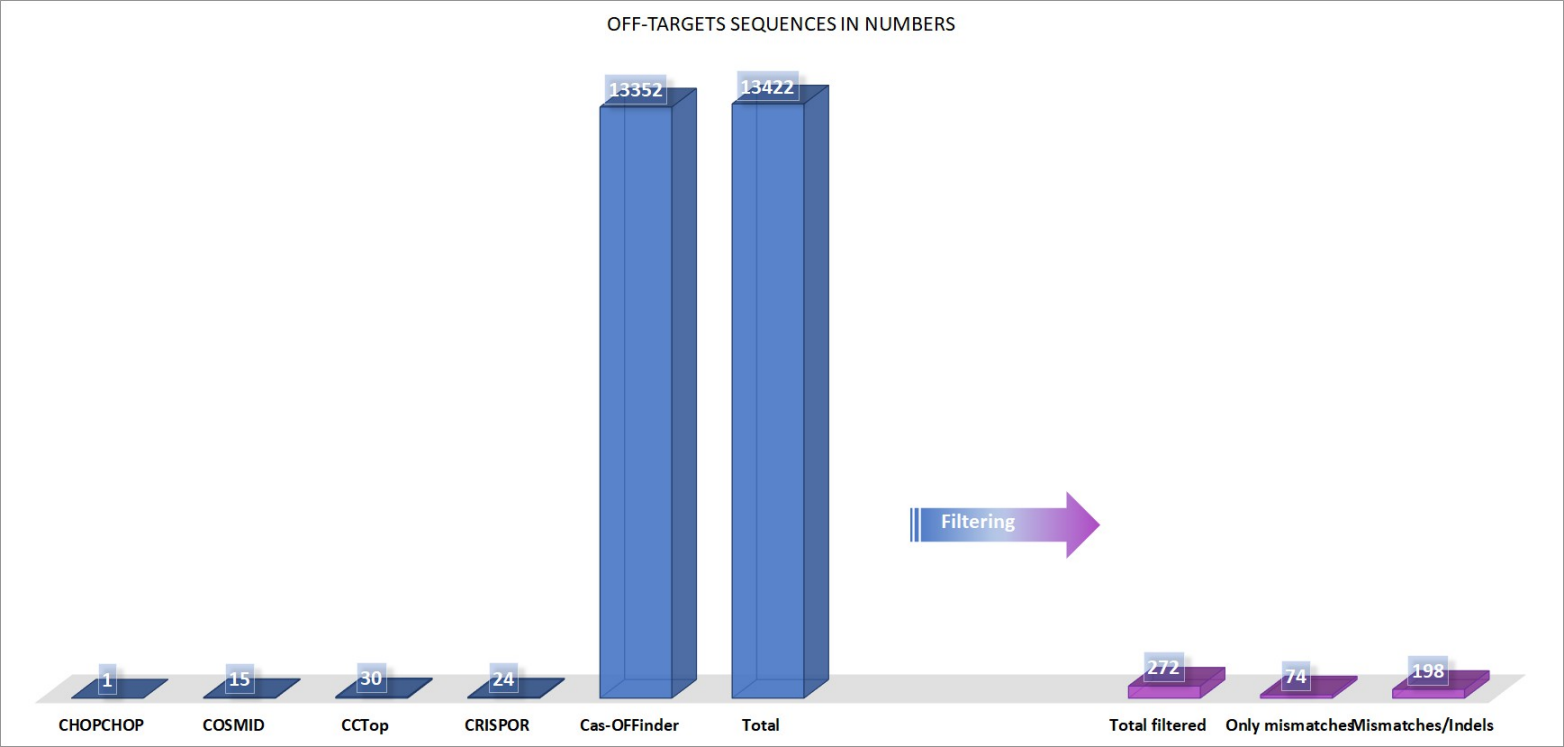
Off-target prediction to p.Trp402* sgRNA by predictors and filter criterias. Graphic illustration of the off-target prediction to p.Trp402* sgRNA before and after the filtering steps. The image shows the number of sequences returned for each of the five predictors and the number of sequences after the filtering step following the methodology’s criteria.

A total of 13,422 off-target sequences with canonical PAM was obtained for the target p.Trp402* sgRNA using the five predictors against the reference human genome. CHOPCHOP and COSMID recognized fewer off-target sites (1 and 15, respectively), whereas CCTop and CRISPOR returned 30 and 24 sequences. Moreover, Cas-OFFinder brought the highest number of sequences with 13,352 predicted off-target sites. In general, the agreement between predictors was very low. Cas-OFFinder and CCTop were the two that showed the highest agreement (26 sequences in common), followed by CRISPOR, Cas-OFFinder, and CCTop, with two (S1 Fig). After filtering for sequences with up to 6 mismatches and up to 2 indels, 272 sequences were obtained as potential off-target sites for this sgRNA, besides the on-target region. Of these sequences, 74 contained only mismatches, and the rest showed both mismatches and indels (Fig 2). Predicted off-target sequences include genic (161) and intergenic regions (111), and a pseudogene. Table 1 shows the top 20 sequences with fewer mismatches or indels, and the complete results can be found in S1 Table. Both tables contain a sequence identification tag used to navigate all other tables in the manuscript.

**Table 1:** Potential off-target regions found by the predictors.

ID	SEQUENCES	M/I	GENOMIC LOCALIZATION	ANNOTATION
0	GCTCTAGGCCGAAGTGTCGCAGG	0/0	chr4:996530–996552	*IDUA*
1	GCTCT**G**GGCCGAAGTGTCGCAGG	1/0	chr4:996530–996552	*IDUA*
2	GCTCT**G**GGC**T**G**GG**GTGTCGCTGG	4/0	chr10:118919279–118919301	*MIR366HG*
3	**T**CTCTAGGC**A**GAAGTG**AT**GCTGG	4/0	chr4:148041025–148041047	Intergenic
4	GCTCTAGGC**T**GAAGTG**CTT**CTGG	4/0	chr8:130569489–130569511	*CCDC26*
5	**C**C**A**CTAGGCC**A**AAGTGT**A**GCTGG	4/0	chr19:8456438–8456460	*RAB11B*
6	GCTC**C**AGG**AG**GAAGTGTC**A**CAGG	4/0	chr3:51437682–51437704	*DCAF1*
7	G**T**TCTAGG**TG**GAAGTGT**T**GCTGG	4/0	chr8:13103641–13103663	*DLC1*
26	G**G**TCTAGGCCAAGCTGTCGCTGG	4/0	chr17:31707604–31707626	*ASIC2*
75	GCTC-AGGC**T**GAAG**G**GTCGCAGG	2/1	chr4:89584514–89584535	*HERC3*
76	GCTCT**G**GG**A**C—GAGTGTCGCTGG	2/1	chr19:7294879–7294900	Intergenic
77	GCTCT**G**-GCCGAAGTG**A**CTCAGG	2/1	chr8:47028210–47028231	Intergenic
78	**A**CTCTA**T**GC**T**GA-GTGTCGCTGG	3/1	chr12:111599505–111599526	*CUX2*
79	G**AA**CTAGGCCG**T**A-TGTCGCTGG	3/1	chr9:90693036–90693057	Intergenic
80	GC**G**CTG-GCCG**C**AG**A**GTCGCCGG	3/1	chrX:153618565–153618586	Intergenic
81	**A**CT-**G**AGG**T**CGAAGTGTCGCTGG	3/1	chr9:125383152–125383173	Intergenic
82	**C**C**C**CTAGGCC**T**AAG-GTCGCGGG	3/1	chr9:95886033–95886054	*NINJ1*
83	GC**G**CT-GGCCG**C**AG**A**GTCGCCGG	3/1	chrX:153618565–153618586	Intergenic
84	GC**G**CT-GGCCG**C**AG**A**GTCGCCGG	3/1	chrX:153570256–153570277	Intergenic
85	GCT**T**TAGG**G**C**C**AAG-GTCGCTGG	3/1	chr18:58811051–58811072	Intergenic

The table shows sequence identification tag (ID) following Table 1, sequence, mismatches (bold) and deletions represented by traverse signal, number of mismatches or insertions (M/I), genomic localization, and off-target annotation to a gene or intergenic region (Annotation).

^a.^Sequence ID follows S1 Table.

The probability of cleavage for these off-target sequences was determined according to CFD score. A total of 15 of these sequences, disregarding the on-target sequence, had a cleavage probability equal or greater than 0.2, which is Doench et al. cut-off values based on experimental observations. These sequences are both in genic (11) and intergenic regions (4). Among these sequences, no one with an indel was recognized with a probability of 0.2 to cleavage. On the other hand, if the cleavage probability threshold of 0.023 is considered, then there are 48 genic and 39 intergenic regions (Table 2 and S2 Table). And in this case, 32 off-target sequences containing indels were included in the list, some of them with CFD scores close to 0.2.

**Table 2:** Probability cleavage of off-target sequences with mismatches and indels.

ID	SEQUENCE	ANNOTATION	CFD	CFD INDEL	CFD FINAL
9	AATCCAGGTTGAAGTGTCGCCGG	Intergenic	0.539	-	0.539 **
2	GCTCTGGGCTGGGGTGTCGCTGG	*MIR366HG*	0.317	-	0.317 **
3	TCTCTAGGCAGAAGTGATGCTGG	Intergenic	0.297	-	0.297 **
4	GCTCTAGGCTGAAGTGCTTCTGG	*CCDC26*	0.285	-	0.285 **
5	CCACTAGGCCAAAGTGTAGCTGG	*RAB11B*	0.275	-	0.275 **
90	ACTCTGAGGCCAAGGTGTCGCAGG	Intergenic	0.419	0.328	0.138 *
132	GCTGCGGACTCCAAGTGTCGCCGG	*LNCOC1*	0.153	0.651	0.100 *
116	GCTCTCAGACCATGGTGTCGCTGG	*ANKRD11*	0.217	0.438	0.095 *
81	ACT-GAGGTCGAAGTGTCGCTGG	Intergenic	0.504	0.176	0.089 *
117	GCTGCAGACACGATGTGTCGCGGG	Intergenic	0.150	0.569	0.085 *

The table shows sequence identification tag (ID) following Table 1, sequence, off-target annotation to genic or intergenic region, CFD to compute mismatches, CFD INDEL to compute indels and CFD FINAL to compute the occurrence of both independent events. CFD INDEL with a dash sign (-) means no score is provided for sequences without indel. The CFD FINAL with ** and * are according to CFD > = 0.2 and CFD > = 0.023, respectively. CFD values are rounded for three decimal places.

**Fig 3:**
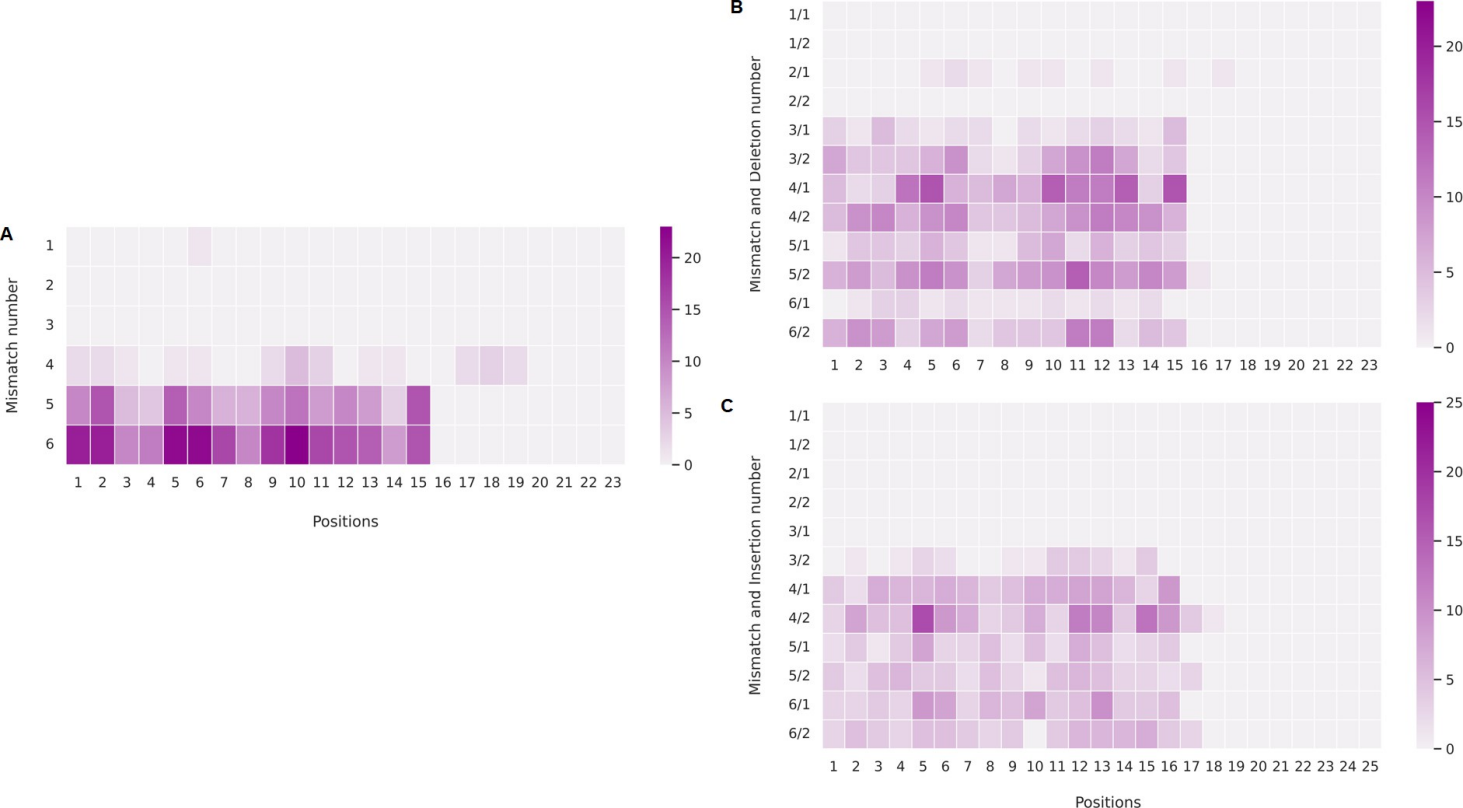
Mismatches and indels positions in off-target sequences. Heatmap of mismatches only (A), deletions plus mismatches (B) and insertions plus mismatches (C) in off-target sequences. Note that, for sequences with mismatches only, PAM is located at positions 21 to 23. In sequences with mismatches and indels, PAM may be located anywhere between positions 19 to 25. The color intensity bar shows the number of sequences with mismatches and/or indels at each given position.

Fig 3 shows the number and distribution of mismatches and indels in off-target sequences. Note that, for sequences with mismatches only, PAM is located at positions 21 to 23. In sequences with mismatches and indels, PAM may be located anywhere between positions 19 to 25. Any off-target sequences with only 2 or 3 mismatches were found. The non-random distribution of mismatch and indel positions can be observed, as most of them are concentrated at positions 1 to 16. In part, this is due to the conservation criteria of SEED and PAM sequences. We cannot discard the possibility that similar off-target sequences found in different genomic regions are responsible for this result. Indeed, twenty-seven identical off-target sequences appeared more than once in different genomic positions.

To investigate if these identical sequences belong to repetitive elements, we used RepeatMasker. Potential off-target sequences were located inside (n = 17) or around (n = 4) repetitive elements (Table 3 and S3 Table).

**Table 3:** Off-target sequences inside repetitive elements.

ID	SEQUENCE	GENOME LOCALIZATION	REPETITIVE ELEMENT
15	AATCCAGGTCGAAGGGTCGCCGG	chr16:3131030–3131052	ERV_classII
16	AATCCAGGTCGAAGGGTCGCCGG	chr10:81735284–81735306	ERV_classII
17	AATCCAGGTCGAAGGGTCGCTGG	chr11:59691555–59691577	ERV_classII
18	AATCCAGGTCGAAGGGTCGCCGG	chr6:67041123–67041145	LINE1/ERV_classII
19	AATCCAGGCTGAAGGGTCGCTGG	chrY:21455475–21455497	ERV_classII

Example of five identical off-target sequences located in different genomic locations inside repetitive elements. The table shows sequence identification tag (ID) following Table 1, genomic localization and repetitive element found by using RepeatMasker.

^a.^Sequence ID follows S1 Table.

**Fig 4:**
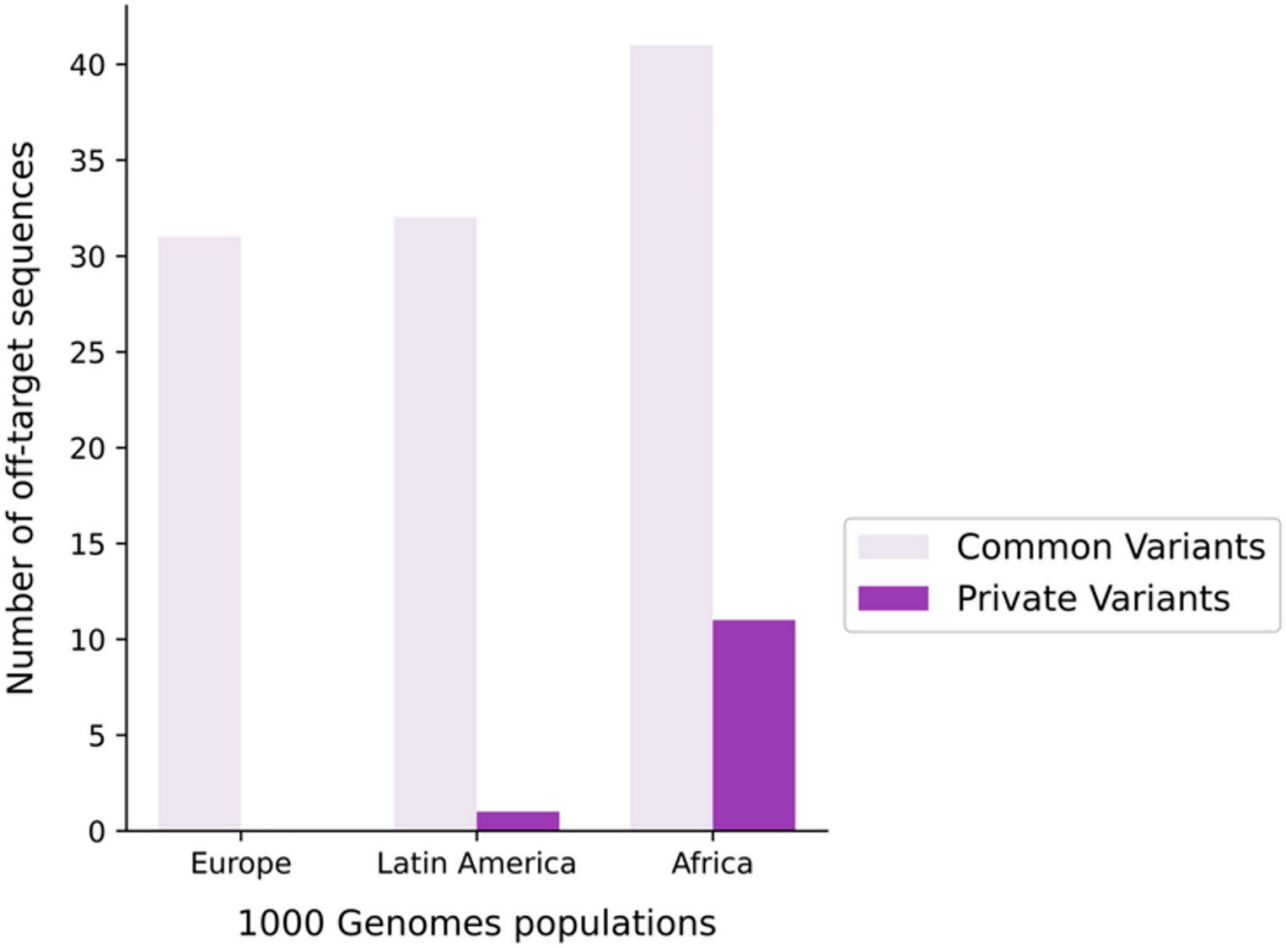
Amount of common and private polymorphic sites for each population. Number of polymorphic sites in off-target sequences in the three populations. The graphic shows the private and shared polymorphic sites with allele frequencies equal to or greater than 1% by using the 1000 Genomes database.

Next, we searched for polymorphic sites in the 271 predicted off-target sequences using population databases. A total of 84 polymorphic sites were recognized in 73 off-target sequences. Most polymorphic sites were found in 1000 Genomes, and only six were exclusive to Latin America, found in ABraOM. LOVD 3.0 Argentina returned no polymorphic sites within these off-target sequences (Fig 4). Although the cut-off value was frequency equal to or greater than 1% in any population, it is worth noticing that some of these variants show a high frequency in many populations (Table 4). A complete list of polymorphic sites and their variant frequencies can be found in S4 and S5 Tables to 1000 Genomes Database and ABraOM, respectively.

**Table 4:** Polymorphic sites in off-target sequences and different allele frequencies among populations.

ID	SEQUENCE	REF	ALT	E	LA	A	W
5	CCACTAGGCCAAAGT**GTA**GCTGG	TAC	T	4.3	3.7	11.3	7.0
75	GCTC-AGGCTGAAGGGT**C**GCAGG	C	T	0	0.6	3.1	0.9
75	GCTC-AGGCTGAA**G**GGTCGCAGG	G	C	0	0	1.4	0.4
82	CCCCTAGGCCTAAG-GTCG**C**GGG	C	T	81.0	87.8	62.9	79.0

Allele frequency of variants in predicted off-target sites shown in Table 1. Allele frequency is shown for three populations (Europe–E, Latin America–LA, Africa–A) and worldwide frequency (–W) according to the 1000 Genomes database. Position of the alternative allele in bold.

Data from the 1000 Genomes database showed that Africa and Latin America have 11 and 1 private polymorphic sites, respectively, not shared with other populations. Europe has no private polymorphic site with a frequency equal to or more than 1% (Fig 4).

We then verified if these polymorphic sites increase or decrease the probability of off-target cleavage by changing the number of mismatches and indels. In most cases, for this sgRNA, the variants decrease the probability of off-target cleavage by increasing the number of mismatches and/or indels. Only six polymorphic variants decrease the number of mismatches in potential off-target sequences (Table 5 and S6 Table).

**Table 5:** Effect of polymorphic sites in off-target sequences in mismatches and indels amount.

ID	SEQUENCE	REF	ALT	NO VARIANT	VARIANT	PAM
**INCREASE MISMATCHES**						
5	CCACTAGGCCAAAGT**GTA**GCTGG	TAC	T	4/0	5/2	-
75	GCTC-AGGCTGAAGGGT**C**GCAGG	C	T	2/1	3/1	-
75	GCTC-AGGCTGAA**G**GGTCGCAGG	G	C	2/1	3/1	-
82	CCCCTAGGCCTAAG-GTCG**C**GGG	C	T	3/1	4/1	-
**DECREASE MISMATCHES**						
44	AATCCAGG**T**TGAAGGGTCGCTGG	T	C	6/0	5/0	-
73	T**T**TCTAGCCAGGACTGTCGCTGG	A	G	6/0	5/0	-
146	GCTCATAAGCAG**G**GCTGTCGCTGG	G	A	5/1	4/1	-
223	GCTCCAGGCCCTG**C**CTTGTCGCTGG	C	A	4/2	3/2	-
227	GCTC**C**CGGGCCC—TGTCGCTGG	G	A	5/2	4/2	-
265	GT**A**CCAGTTC—AGGGTCGCAGG	T	A	6/2	5/2	-

The table shows sequence identification tag (ID) following Table 1, the sequence with the alternative allele in bold, reference and alternative alleles, and the number of mismatches and indels without (no variant) or with the variant (variant). Off-target sequences are shown in Table 1, and the six other off-target sequences with a decrease in the number of mismatches are depicted.

Furthermore, six other polymorphic sites disrupt the PAM sequence, hence decreasing the probability of off-target cleavage. However, such variants’ frequency is different among populations, and their impact must be considered in a population-specific context (Table 6). Also, only one polymorphic site with a frequency greater than 1% created a new PAM for this sgRNA (Table 6).

**Table 6:** Polymorphic sites disrupt existing canonical PAMs and create new ones.

ID	SEQUENCE	REF	ALT	PAM	E	LA	A	W
**DISRUPTED**	**PAM**							
9	AATCCAGGTTGAAGTGTCGCC**G**G	C	T	CAG	22.3	17.7	2.7	11.6
18	AATCCAGGTCGAAGGGTCGCC**G**G	C	T	CAG	0.1	0.4	6.1	1.7
59	GCTCTGCCCTGAGGGGTCGCAG**G**	C	A	AGT	0	1.0	9.2	2.6
97	CCTCTAGACC-AGGGGTCGCA**G**G	G	A	AAG	0	1.1	0	0.7
123	GTTCTTAGTCCAATGTGTCGCC**G**G	C	T	CAG	0	0.3	2.9	0.8
173	GCTGTTTGCC—AGTGTCGCT**G**G	G	A	TAG	0	0.6	3.9	1.1
**CREATED**	**PAM**							
273	GCTTATGCCAGTAGTGTCGCAG**T**	T	G	AGG	16.0	16	42.3	24.8

The table shows sequence identification tag (ID) following Table 1, the sequence with the alternative allele in bold, reference and alternative alleles, new PAM sequence formed, and frequency of the alternative allele in each of the three populations (Europeans–E, Latin Americans—LA, Africans–A) and worldwide frequency (–W) according to the 1000 Genomes database.

^a.^Sequence ID follows S1 Table.

## Discussion

Software developed to predict CRISPR editing tools’ effects, include the design of sgRNA, the calculated efficiency, and potential off-target sites. *In silico* prediction of off-target sites requires parameters that were shown to be important in experimental assays. The canonical PAM sequence (NGG) from *Streptococcus pyogenes* and the number and position of mismatches are some default criteria that are always considered in the search. Even so, tools diverge in other parameters and hence provide different results according to the used algorithm.

In this investigation of off-target sites for a sgRNA for a monogenic disease, we chose the five most used *in silico* predictors in the literature [17]. The Cas-OFFinder was the tool that brought more off-target sequences, but together only 272 sequences were selected as potential off-target sites, limited by the preservation of the 5 PAM adjacent nucleotides and up to 6 and 2 mismatches and indels, respectively. Out of these, 112 potential off-target sequences, including the one sequence with new PAM, are in intergenic regions. Following the CFD score, we showed that for some off-targets our sgRNA has a considerable probability of cleavage for at least 15 off-target sequences. In addition, sequences with indel had a CFD final score equal or greater than 0.023 but none passed the 0.2 threshold. The sequence with indel that has a maximum CFD final score is approximately 0.14. As expected, the indels inside these sequences were located at least 7 nt distal to PAM, but these may be due to SEED selection. Nevertheless, these results indicate that sequences with less or greater length than 23 nt and indels distal to PAM could be cut by the sgRNA during gene editing.

Some similar sequences in different genomic regions are localized inside repeat elements as transposable elements (TE). Although counter-intuitive, given the TE’s mobile nature, recently, it was shown that they are involved in maintaining 3D genome architecture [32]. The deleterious effect of disrupting non-coding regions, regulatory elements, and TEs involved in 3D genome structure integrity must be considered. The evaluation of the biological context to which these sequences belong will help assess their possible off-target effects in the human genome. None of the software used considers chromatin level information, despite acknowledging its importance. Experimental assays showed that nucleosomes interfere with CRISPR/Cas9 cleavage in fungi strains [33]. In that sense, cell type may also be an essential factor, as it affects chromatin architecture and the relative abundance of repair mechanisms.

This evaluation investigated if the tool’s output could be altered by genetic variability between different populations. We found that approximately 27% (n = 73) of the 272 potential off-target sequences, including the one with new PAM, have polymorphic sites with a frequency greater than 1% in at least one of the three considered populations (Europe, Africa, and Latin America) according to the 1000 Genomes database and ABraOM databases. In some of these sequences, more than one polymorphic site was found. These polymorphic sites may interfere in the number of mismatches, especially in SEED and PAM, and influence the actual cleavage. Approximately 15% of variants (n = 40) in the 272 predicted potential off-target sites disrupt PAM and/or SEED. Also, six variants decrease the number of mismatches, and one can create a new PAM for the sgRNA. Moreover, the variant creating this new PAM sequence has a frequency of 42% in populations from Africa and 16% in populations from Europe and Latin America. Altogether, we found six polymorphic sites in 5 sequences that were only identified by the ABraOM database, which highlights the importance of population diversity in databases. Furthermore, the genetic variability evaluation showed that some alternative alleles in specific populations or worldwide are more frequent than the reference allele (S4 and S5 Tables). This analysis shows the importance of population variability in off-target analyses. Despite that, all the five most used tools do not take into account this variability in their off-target evaluation. As an indication that this is indeed a relevant question, some recently developed bioinformatic tools include genetic variability [34, 35]. However, these tools have limitations. For instance, CrisPam needs the nucleotide variant identifier to detect the new PAM, which is limited compared to our approach of screening for any possible variant creating a new PAM and polymorphic sites within the off-target sequences that could influence cleavage probability. Also, both tools rely on dbSNP as the sole database, whereas we used different population-based databases to find information about variant frequency and private variants in some populations.

The improvement of genome editing methods raises other therapeutic alternatives by using base editing techniques for treatment of diseases that are a consequence of a single variant. In this view, the most common variant found in MPS type I is a G to A transition that could benefit from the adenine base editor genome editing technique as treatment [36]. Although this technique also presents off-target concerns and efficiency differences according to the modifications [37, 38], it could benefit patients’ treatments. Even so, the presence of polymorphic sites within sequences could still compromise the safety of patients from particular populations, although to a lesser extent.

In this work, we used as a model the sgRNA designed to correct the most common pathogenic variant found in MPS I patients across different populations. We developed a comprehensive approach to identify potential off-target sites considering the standard output of *in silico* predictors combined with analysis of frequent polymorphic sites in different populations by using three different human variation databases. In general, the recognition of polymorphic sites leads to a decrease in the chance of off-target cleavage by CRISPR-Cas9. In this sense, this analysis contributes to a better evaluation of gene editing safety aspects, and relevant differences are found among populations for MPS I patients’ treatment, which to the extent of our knowledge, is the first attempt to address this question. Therefore, we recommend that such an approach be implemented in off-target prediction for each disease, considering the specific population for which it is designed, complemented by experimental validation. As CRISPR system therapies enter the clinical arena, we must ensure their safe use in different world regions.

## Supporting information

S1 Fig Venn diagram for potential off-target sites returned by each of the five predictors used. All predictors detected the wild-type target sequence as an off-target site. Venn diagram made using InteractiVenn.
(PNG)

S1 Table Potential off-target regions found by the five predictors. The table shows sequence identification tag (ID), sequence, mismatches (bold) and indels (insertions in italic and deletions represented by traverse signal), number of mismatches or insertions (M/I), genomic localization, and off-target annotation to a gene or intergenic region (Annotation).
(DOCX)

S2 Table The full CFD final score evaluation for off-target sequences. The table shows the cleavage probability for each off-target sequence (with identification tag (ID) following Table 1), off-target annotation, CFD, CFD INDEL and CFD FINAL for all possible off-target sequences returned by the predictors and BWA alignment sequence (ID 273). CFD INDEL with a dash sign means no score was provided for sequences without indel. The CFD FINAL with ** and * are according to CFD > = 0.2 and CFD > = 0.023, respectively.
(DOCX)

S3 Table Off-target sequences located in different genomic locations inside repetitive elements. The table shows sequence identification tag (ID), sequence, genomic localization and the repeat element where signal (-) and signal (*) denote no repeat element found or sequences close to repeat elements in the region evaluated, respectively.
(DOCX)

S4 Table Allele frequency of variants in predicted off-target sites shown in Table 1. Allele frequency is shown for three populations (Europe–E, Latin America–LA, Africa–A) and worldwide frequency (–W) according to the 1000 Genomes database. Position of the alternative allele in bold.
(DOCX)

S5 Table Allele frequency of variants in predicted off-target sites shown in Table 1. Allele frequency is shown for the Brazilian population according to the ABraOM database. Position of the alternative allele in bold. The star signal (*) denotes variants not found in the 1000 Genomes database.
(DOCX)

S6 Table Effect of polymorphic sites in off-target sequences. The table shows sequence identification tag (ID) following Table 1, the sequence with the alternative allele in bold, reference and alternative alleles, and the number of mismatches and indels without (no variant) or with the variant (variant).
(DOCX)

S1 File CFD indel score program. This program accounts only indels in a specific sequence by giving the cRNA with insertion and off-target with deletions positions for sequences greater or less than 23 nt, respectively, denoted by dash sign (-). The first parameter is the cRNA and the second is off-target sequence. Besides, it removes only the insertion from a specific sequence.
(ZIP)
