# Supplementary figures and images for: In silico analysis of potential off-target sites to gene editing for Mucopolysaccharidosis type I using the CRISPR/Cas9 system: Implications for population-specific treatments
Source: PLoS One. 2022 Jan 24;17(1):e0262299. doi: 10.1371/journal.pone.0262299 (PMC8786118; doi:10.1371/journal.pone.0262299)

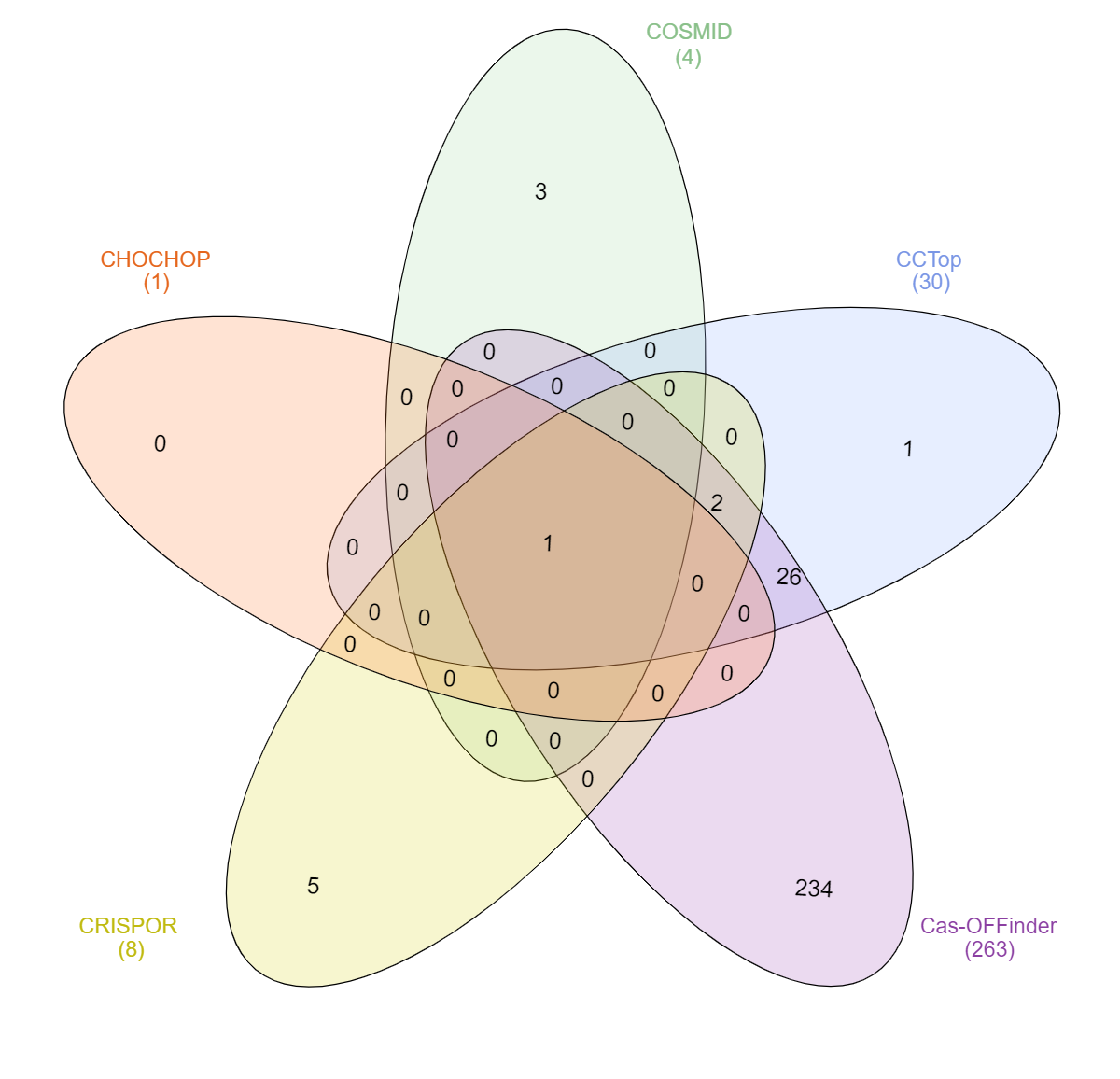

Supplement: S1 Fig — All predictors detected the wild-type target sequence as an off-target site. Venn diagram made using InteractiVenn. (PNG) [file pone.0262299.s001.png]
