# Supplementary material for: In silico analysis of potential off-target sites to gene editing for Mucopolysaccharidosis type I using the CRISPR/Cas9 system: Implications for population-specific treatments
Source: PLoS One. 2022 Jan 24;17(1):e0262299. doi: 10.1371/journal.pone.0262299 (PMC8786118; doi:10.1371/journal.pone.0262299)
Supplement: S1 Table — The table shows sequence identification tag (ID), sequence, mismatches (bold) and indels (insertions in italic and deletions represented by traverse signal), number of mismatches or insertions (M/I), genomic localization, and off-target annotation to a gene or intergenic region (Annotation). (DOCX) [file pone.0262299.s002.docx]

**S1 Table**

| **ID** | **SEQUENCES** | **M/I** | **GENOMIC LOCALIZATION** | **ANNOTATION** |
| --- | --- | --- | --- | --- |
| 0 | GCTCTAGGCCGAAGTGTCGCAGG | 0/0 | chr4:996530-996552 | *IDUA* |
| 1 | GCTCT**G**GGCCGAAGTGTCGCAGG | 1/0 | chr4:996530-996552 | *IDUA* |
| 2 | GCTCT**G**GGC**T**G**GG**GTGTCGCTGG | 4/0 | chr10:118919279-118919301 | *MIR366HG* |
| 3 | **T**CTCTAGGC**A**GAAGTG**AT**GCTGG | 4/0 | chr4:148041025-148041047 | Intergenic |
| 4 | GCTCTAGGC**T**GAAGTG**CTT**CTGG | 4/0 | chr8:130569489-130569511 | *CCDC26* |
| 5 | **C**C**A**CTAGGCC**A**AAGTGT**A**GCTGG | 4/0 | chr19:8456438-8456460 | *RAB11B* |
| 6 | GCTC**C**AGG**AG**GAAGTGTC**A**CAGG | 4/0 | chr3:51437682-51437704 | *DCAF1* |
| 7 | G**T**TCTAGG**TG**GAAGTGT**T**GCTGG | 4/0 | chr8:13103641-13103663 | *DLC1* |
| 8 | G**G**T**A**T**CA**GC**A**GAAGTGTCGCTGG | 5/0 | chr5:146345164-146345186 | *PPPR2B* |
| 9 | **AA**TC**C**AGG**TT**GAAGTGTCGCCGG | 5/0 | chr12:39594468-39594490 | Intergenic |
| 10 | **C**C**A**C**C**AGGC**T**G**C**AGTGTCGCAGG | 5/0 | chr6:79353909-79353931 | Intergenic |
| 11 | CCACCAGGCTGCAGTGTCGCAGG | 5/0 | chr10:12856342-12856364 | *CAMK1D* |
| 12 | GC**G**C**CC**GGCC**CG**AGTGTCGCGGG | 5/0 | chr7:25991151-25991173 | Intergenic |
| 13 | GCTC**C**A**CA**C**A**G**T**AGTGTCGCGGG | 5/0 | chr1:1151358-1151380 | Intergenic |
| 14 | G**A**T**GG**AGGCCG**CT**GTGTCGCAGG | 5/0 | chr1:180223446-180223468 | *LHX4* |
| 15 | **AA**TC**C**AGG**T**CGAAG**G**GTCGCCGG | 5/0 | chr16:3131030-3131052 | *IL32* |
| 16 | **AA**TC**C**AGG**T**CGAAG**G**GTCGCCGG | 5/0 | chr10:81735284-81735306 | *SFTPD* |
| 17 | **AA**TC**C**AGG**T**CGAAG**G**GTCGCTGG | 5/0 | chr11:59691555-59691577 | Intergenic |
| 18 | **AA**TC**C**AGG**T**CGAAG**G**GTCGCCGG | 5/0 | chr6:67041123-67041145 | Intergenic |
| 19 | **AA**TC**C**AGGC**T**GAAG**G**GTCGCTGG | 5/0 | chrY:21455475-21455497 | Intergenic |
| 20 | **TT**TCTAGGC**TC**A**C**GTGTCGCTGG | 5/0 | chr19:5371949-5371971 | Intergenic |
| 21 | G**G**TC**A**A**CC**CCGAAG**A**GTCGCCGG | 5/0 | chr8:29120598-29120620 | *KIF13B* |
| 22 | GC**C**CT**G**G**T**CCG**CT**GTGTCGCTGG | 5/0 | chr17:63052331-63052353 | *GNA13* |
| 23 | GCTC**G**AG**AT**C**A**A**G**GTGTCGCAGG | 5/0 | chr11:70689763-70689785 | *SHANK2* |
| 24 | G**A**TCT**G**GG**GA**GAAG**G**GTCGCAGG | 5/0 | chr19:50427945-50427967 | *IL4I1* |
| 25 | **CT**TC**C**AGGCCGA**G**G**A**GTCGCCGG | 5/0 | chr9:132640628-132640650 | *USP20* |
| 26 | G**G**TCTAGGCC**A**A**GC**TGTCGCTGG | 4/0 | chr17:31707604-31707626 | *ASIC2* |
| 27 | GC**G**CT**G**GGC**G**G**G**A**T**TGTCGCAGG | 5/0 | chr7:155601333-155601355 | *SHH* |
| 28 | GCT**T**T**G**GGC**G**G**C**AG**G**GTCGCAGG | 5/0 | chr19:2307987-2308009 | *LINGO3* |
| 29 | G**T**TCT**T**GG**G**CGA**T**G**A**GTCGCGGG | 5/0 | chr6:33281749-33281771 | *TAPBP* |
| 30 | GCT**GA**AGGCC**TG**AG**G**GTCGCCGG | 5/0 | chr15:83777349-83777371 | *TM6SF1* |
| 31 | GCTCTA**AT**C**AT**AAG**C**GTCGCAGG | 5/0 | chr16:47497815-47497837 | *PHKB* |
| 32 | GC**A**CTA**CA**CCGAA**AC**GTCGCGGG | 5/0 | chr20:42086382-42086404 | Intergenic |
| 33 | GCTC**G**AG**C**CC**AG**AG**G**GTCGCCGG | 5/0 | chr5:39073917-39073939 | *RICTOR* |
| 34 | G**A**TCTAGGC**TA**A**G**G**A**GTCGCAGG | 5/0 | chr3:126655025-126655047 | *CHCHD6* |
| 35 | GCTCTA**A**GC**T**G**GCC**TGTCGCTGG | 5/0 | chr2:220489550-220489572 | Intergenic |
| 36 | **A**CT**G**TAG**T**CC**T**A**CA**TGTCGCTGG | 6/0 | chr8:13730106-13730128 | Intergenic |
| 37 | GCTC**CTCCAG**GAAGTGTCGCAGG | 6/0 | chr8:23288368-23288390 | *ENTPD4* |
| 38 | **AT**T**T**T**T**GGC**A**G**C**AGTGTCGCTGG | 6/0 | chr12:16574919-16574941 | *MGST1* |
| 39 | **T**CTCT**TT**G**G**C**A**AAG**G**GTCGCTGG | 6/0 | chr12:46080385-46080407 | Intergenic |
| 40 | **TG**TC**CTT**GC**T**GAAGTGTCGCAGG | 6/0 | chr12:119259651-119259673 | *LINC02439* |
| 41 | **TT**TCT**CC**G**T**CG**C**AGTGTCGCAGG | 6/0 | chr12:125877331-125877353 | *TMEM132B* |
| 42 | G**TG**CT**G**GGCC**T**A**TT**TGTCGCTGG | 6/0 | chr3:87409315-87409337 | Intergenic |
| 43 | GCTCT**TCTG**C**C**A**G**GTGTCGCTGG | 6/0 | chr3:108156408 -108156430 | *MYH15* |
| 44 | **AA**TC**C**AGG**TT**GAAG**G**GTCGCTGG | 6/0 | chr3:184823526-184823548 | *C3orf70* |
| 45 | GC**C**CTAGGC**AT**A**TCA**GTCGCTGG | 6/0 | chr3:193952534-193952556 | *LIN02036* |
| 46 | GCTC**CTCTA**CcAAGTGTCGCAGG | 6/0 | chr7: 334104-334126 | Intergenic |
| 47 | GC**C**C**C**A**A**G**TA**GAAG**G**GTCGCAGG | 6/0 | chr7:534830-534852 | Intergenic |
| 48 | **C**CTC**GTC**GCCG**CT**GTGTCGCTGG | 6/0 | chr7:1105256-1105278 | *C7orf50* |
| 49 | G**T**T**AA**AG**AA**C**A**AAGTGTCGCTGG | 6/0 | chr7: 3678772-3678794 | *SDK1* |
| 50 | GC**C**CT**C**G**T**CCG**TCT**TGTCGCAGG | 6/0 | chr7:27195844-27195866 | *HOXA7* |
| 51 | **C**CT**G**T**TAT**C**A**GAAGTGTCGCAGG | 6/0 | chr7:44106164 -44106186 | *DBNL* |
| 52 | G**GA**CT**GT**G**GG**GAAGTGTCGCGGG | 6/0 | chr7:55639474-55639496 | *VOPP1* |
| 53 | **CAC**C**C**AGGC**T**G**G**AGTGTCGCGGG | 6/0 | chr7: 57129464-57129486 | Intergenic |
| 54 | **A**CTCT**G**GGCC**AGGC**TGTCGCAGG | 6/0 | chr7:140924451-140924473 | *TMEM178B* |
| 55 | G**A**TC**A**AGGC**TACT**GTGTCGCTGG | 6/0 | chr4:4664672-4664694 | Intergenic |
| 56 | **A**CT**ACC**GGC**A**G**G**AGTGTCGCCGG | 6/0 | chr4: 7968537-7968559 | *ABLIM2* |
| 57 | **A**CTCT**GT**GC**TC**A**T**GTGTCGCAGG | 6/0 | chr4:38271835-38271857 | Intergenic |
| 58 | **ATC**CTAGGCC**CCT**GTGTCGCAGG | 6/0 | chr4:114864472-114864494 | *ARJS* |
| 59 | GCTCT**GCC**C**T**GA**G**G**G**GTCGCAGG | 6/0 | chr5:11123042-11123064 | *CTNND2* |
| 60 | **TG**T**TC**AGGCC**TC**AGTGTCGCTGG | 6/0 | chr5:42461909 -42461931 | *GHR* |
| 61 | **AA**TC**C**AGG**TT**GAAG**A**GTCGCTGG | 6/0 | chr5:44986557-44986579 | Intergenic |
| 62 | **A**CT**GC**AGG**TG**GAA**A**TGTCGCAGG | 6/0 | chr5:79133696 – 79133718 | Intergenic |
| 63 | G**A**T**GC**A**A**G**AG**GAAGTGTCGCTGG | 6/0 | chr5:94445982-94446004 | *MCTP1* |
| 64 | **CT**TC**CG**GGCCG**C**AG**G**GTCGCGGG | 6/0 | chr5:167956264-167956286 | *FBLL1* |
| 65 | GC**C**CT**CA**G**G**C**A**AAG**A**GTCGCAGG | 6/0 | chr8:91233920 -91233942 | *LINC00534* |
| 66 | GCTC**CT**GGC**G**G**TT**G**C**GTCGCTGG | 6/0 | chr8:144523132-144523154 | *ZC3H3* |
| 67 | G**G**T**TG**AGG**TG**GAAG**A**GTCGCTGG | 6/0 | chr7:29967636-29967658 | *SCRN1* |
| 68 | G**TC**C**A**AGGC**A**G**G**AG**G**GTCGCTGG | 6/0 | chr7:129998073-129998095 | *CPA5* |
| 69 | G**GA**C**C**AGGC**G**GA**G**G**C**GTCGCGGG | 6/0 | chr7:148396072-148396094 | *CUL1* |
| 70 | **AA**TC**C**AGG**T**C**A**AAG**G**GTCGCTGG | 6/0 | chr4: 45044604-45044626 | Intergenic |
| 71 | **AA**TC**C**AGG**TT**GAAG**G**GTCGCTGG | 6/0 | chr4:98023220-98023242 | Intergenic |
| 72 | GCTCT**CT**G**A**C**C**AA**CA**GTCGCAGG | 6/0 | chr4:117519558-117519580 | Pseudogene |
| 73 | **TT**TCTAG**C**C**A**G**G**A**C**TGTCGCTGG | 6/0 | chr4:124387120-124387141 | Intergenic |
| 74 | GCT**AA**A**A**G**T**C**A**A**G**GTGTCGCAGG | 6/0 | chr5:97172700-97172722 | Intergenic |
| 75 | GCTC-AGGC**T**GAAG**G**GTCGCAGG | 2/1 | chr4:89584514-89584535 | *HERC3* |
| 76 | GCTCT**G**GG**A**CG-AGTGTCGCTGG | 2/1 | chr19:7294879-7294900 | Intergenic |
| 77 | GCTCT**G**-GCCGAAGTG**A**CTCAGG | 2/1 | chr8:47028210-47028231 | Intergenic |
| 78 | **A**CTCTA**T**GC**T**GA-GTGTCGCTGG | 3/1 | chr12:111599505 - 111599526 | *CUX2* |
| 79 | G**AA**CTAGGCCG**T**A-TGTCGCTGG | 3/1 | chr9:90693036 – 90693057 | Intergenic |
| 80 | GC**G**CTG-GCCG**C**AG**A**GTCGCCGG | 3/1 | chrX:153618565-153618586 | Intergenic |
| 81 | **A**CT-**G**AGG**T**CGAAGTGTCGCTGG | 3/1 | chr9:125383152-125383173 | Intergenic |
| 82 | **C**C**C**CTAGGCC**T**AAG-GTCGCGGG | 3/1 | chr9:95886033-95886054 | *NINJ1* |
| 83 | GC**G**CT-GGCCG**C**AG**A**GTCGCCGG | 3/1 | chrX:153618565-153618586 | Intergenic |
| 84 | GC**G**CT-GGCCG**C**AG**A**GTCGCCGG | 3/1 | chrX:153570256-153570277 | Intergenic |
| 85 | GCT**T**TAGG**G**C**C**AAG-GTCGCTGG | 3/1 | chr18:58811051-58811072 | Intergenic |
| 86 | GCTCTAG**TA**C**AG**-GTGTCGCTGG | 4/1 | chr7:33754054-33754075 | Intergenic |
| 87 | G**A**TCTA**TA**CC**C**A-GTGTCGCTGG | 4/1 | chr7:75049729-75049750 | *POM121C* |
| 88 | **T**CTCT**T**GG**G**C**C**A-GTGTCGCAGG | 4/1 | chr4: 182426111-182426132 | Intergenic |
| 89 | GC**AA**TAG**AG**-GAAGTGTCGCTGG | 4/1 | chr1: 229832258-229832279 | Intergenic |
| 90 | **A**CTCT**G**AGG*C*C**A**A**G**GTGTCGCAGG | 4/1 | chr8:105318003-105318026 | Intergenic |
| 91 | GC**A**C*G*T**G**GGCCGA**G**G**G**GTCGCGGG | 4/1 | chr8:48920856-48920879 | Intergenic |
| 92 | GCT*T*C**CG**GGCCG**C**AG**G**GTCGCGGG | 4/1 | chr5:167956263-167956286 | *FBLL1* |
| 93 | GC**G**C**GG**GGCC*A*GAAG**G**GTCGCGGG | 4/1 | chr1: 19283072-19283095 | *IFFO2* |
| 94 | GCT**A**T**G**GGC**A**GA**G**G*C*TGTCGCTGG | 4/1 | chr15:62119936 -62119959 | Intergenic |
| 95 | GCTC**C**A**C**GC**T**GA**T**G-GTCGCTGG | 4/1 | chr8: 1719291-1719312 | *CLN8* |
| 96 | **C**CTCT**TCA**CC-AAGTGTCGCAGG | 4/1 | chr12: 56031578-56031599 | *OR10P1* |
| 97 | **C**CTCTAG**A**CC-A**G**G**G**GTCGCAGG | 4/1 | chr12:111419398-111419419 | Intergenic |
| 98 | G**G**TCTAG**C**C**T**GA-G**G**GTCGCAGG | 4/1 | chr12:50366554 -50366575 | *AQP6* |
| 99 | GC**A**C**GC**GGCC-A**G**GTGTCGCCGG | 4/1 | chr3:13324291 -13324312 | Intergenic |
| 100 | GC**G**CTAGG**AT**-A**C**GTGTCGCGGG | 4/1 | chr3:139063126 -139063147 | *MRPS22* |
| 101 | GCT**G**-AGGC**A**G**G**AG**A**GTCGCTGG | 4/1 | chr3:88900563-88900584 | Intergenic |
| 102 | **C**CTCTAGGC**A**G**GT**GT*G*GTCGCTGG | 4/1 | chr7:153634111-153634134 | *DPP6* |
| 103 | GCT-**GG**GGC**A**GA**G**GTGTCGCTGG | 4/1 | chr7:5002692-5002713 | Intergenic |
| 104 | GCT-**G**AGGC**A**G**G**AG**A**GTCGCTGG | 4/1 | chr7:6037811-6037832 | *PMS2* |
| 105 | GCT-**G**AGGC**AC**AAG**A**GTCGCTGG | 4/1 | chr7:138367611-138367632 | *SVOPL* |
| 106 | GCTC**G**AGGCC-**GT**G**C**GTCGCGGG | 4/1 | chr7:5229825-5229846 | *WIPI2* |
| 107 | GCT-**G**AGGC**A**G**G**AG**A**GTCGCTGG | 4/1 | chr4:38211033 -38211054 | Intergenic |
| 108 | GCT-**G**AGGC**A**G**G**AG**A**GTCGCTGG | 4/1 | chr4:173419240-173419261 | *GALNTL6* |
| 109 | **A**CTCTA**T**GCC-A**G**G**C**GTCGCTGG | 4/1 | chr4:183062740-183062761 | Intergenic |
| 110 | GCT-T**T**GGC**A**G**G**AG**G**GTCGCTGG | 4/1 | chr5: 7475123-7475144 | *ADCY2* |
| 111 | G**AG**CTAGGCCGA**T**G**G***T*GTCGCTGG | 4/1 | chr16:12553775-12553798 | *SNX29* |
| 112 | GC**AG**TAGGCC**CG**AGT*C*GTCGCCGG | 4/1 | chr16: 54962893-54962916 | *CRNDE* |
| 113 | GCT**A**-AGGC**A**G**G**AG**G**GTCGCTGG | 4/1 | chr16:81123920-81123941 | *GCSH* |
| 114 | GCTC-AG**C**CC**T**A**GC**TGTCGCTGG | 4/1 | chr16:88812549-88812570 | *PIEZO1* |
| 115 | GCTCT-GG**GG**GA**T**G**A**GTCGCAGG | 4/1 | chr16: 83488997-83489018 | *CDH13* |
| 116 | GCTCT*C*AG**A**CC**ATG**GTGTCGCTGG | 4/1 | chr16:89361068-89361091 | *ANKRD11* |
| 117 | GCT**GC**AG**A**C*A*CGA**T**GTGTCGCGGG | 4/1 | chr1:92012573-92012596 | Intergenic |
| 118 | GCTC**C**AGGC*T*CG**GC**G**G**GTCGCGGG | 4/1 | chr1: 206858414-206858437 | *MAPKAPK2* |
| 119 | GCT-**C**AGGC**A**G**T**A**A**TGTCGCCGG | 4/1 | chr1:216379864-216379885 | *USH2A* |
| 120 | GCT-**G**AGGC**A**G**G**AG**A**GTCGCTGG | 4/1 | chr1:233151337-233151358 | *PCNX2* |
| 121 | GCT**GG**A-GCCGA**G**G**C**GTCGCAGG | 4/1 | chr1:224924843-224924864 | *CNIH3* |
| 122 | **C**CTC**CG**GGCCGA**G**G-GTCGCGGG | 4/1 | chr1:211688848-211688869 | Intergenic |
| 123 | G**T**TC*T*TAG**T**CC**A**A**T**GTGTCGCCGG | 4/1 | chr2:235907810-235907833 | *SH3BP4* |
| 124 | GCT**T**T**T**G**AT**CG*T*AAGTGTCGCTGG | 4/1 | chr19:906684-906707 | *R3HDM4* |
| 125 | GCTC**C**AGG**AG***A*GAAG**G**GTCGCTGG | 4/1 | chr17: 2732737-2732760 | *RAP1GAP2* |
| 126 | G**G**TCT*C*AGGC**GC**A**G**GTGTCGCGGG | 4/1 | chr10:102586647-102586670 | *PAX2* |
| 127 | GCTCTA*C***A**GC**A**G**G**AG**G**GTCGCGGG | 4/1 | chr14:73360453-73360476 | *DPF3* |
| 128 | GC*G*T**GA**AGGC**G**G**C**AGTGTCGCCGG | 4/1 | chr9:97847415-97847438 | *AOPEP* |
| 129 | **C**C**C**CTA**A**GC**T**G*C*AAGTGTCGCTGG | 4/1 | chrX:118942357-118942379 | Intergenic |
| 130 | **C**C**C**CTA**A**GC**T**G*C*AAGTGTCGCTGG | 4/1 | chrX: 118950255-118950278 | Intergenic |
| 131 | **T**CTC**C**AGGC*C*C**CC**AG**C**GTCGCAGG | 5/1 | chr8:142678407-142678430 | Intergenic |
| 132 | GCT**GCG**G**A**C*T*C**C**AAGTGTCGCCGG | 5/1 | chr8:143807958 -143807981 | Intergenic |
| 133 | G**G**T-**G**AGG**GA**G**G**AGTGTCGCTGG | 5/1 | chr8:22590914-22590935 | *PEBP4* |
| 134 | GCT-**G**AGG**GA**GA**CT**TGTCGCAGG | 5/1 | chr8:82084024-82084045 | Intergenic |
| 135 | GC**C**-T**G**GGC**A**G**C**A**T**TGTCGCAGG | 5/1 | chr8:144296618 -144296642 | *GPIHBP1* |
| 136 | **A**CTCT**GA**G**G**C**C**AAG*G*TGTCGCAGG | 5/1 | chr8:105318003 -105318026 | Intergenic |
| 137 | GC**C**C**CT**GGCC-A**CC**TGTCGCGGG | 5/1 | chr8:143362093-143362114 | *TSNARE1* |
| 138 | G**G**TC-AGG**TTT**AAG**G**GTCGCTGG | 5/1 | chr8:6565574-6565595 | Intergenic |
| 139 | GC-C**A**AGG**TG**G**G**AG**G**GTCGCTGG | 5/1 | chr8:42046376 -42046397 | *PLAT* |
| 140 | G**A**-CT**T**GGC**T**G**C**AG**A**GTCGCAGG | 5/1 | chr8:68345101-68345122 | *CPA6* |
| 141 | **CA**TC**A**A-GC**T**G**C**AGTGTCGCAGG | 5/1 | chr8:57850246-57850267 | Intergenic |
| 142 | GCTCT-G**TT**CG**TGC**TGTCGCTGG | 5/1 | chr8:2085298 -2085319 | *MYOM2* |
| 143 | GCT**GC**AGGC**G**G**G**AG**G***G*GTCGCGGG | 5/1 | chr12:57940715-57940738 | *DCNT2* |
| 144 | G**G**T**TC**AG**A**CC**C**AAGT*A*GTCGCAGG | 5/1 | chr3:32962577-32962600 | Intergenic |
| 145 | GC**G**C**ATA**GCCG**T**AGT*C*GTCGCAGG | 5/1 | chr3:47459858 -47459881 | *SCAP* |
| 146 | GCTC*A*TA**A**GC**A**G**GGC**TGTCGCTGG | 5/1 | chr3:13262574-13262597 | Intergenic |
| 147 | G**A**T**TC**AGG*T*CCG**C**AG**G**GTCGCTGG | 5/1 | chr3:52869121-52869144 | *STIMATE-MUSTN1* |
| 148 | G**G**TCTA**CA**C*C*C**AC**AGTGTCGCTGG | 5/1 | chr7:72415099-72415122 | *POM121* |
| 149 | G**G**TCTA**CA**C*C*C**AC**AGTGTCGCTGG | 5/1 | chr7:72713486-72713509 | *POM121B* |
| 150 | GCTCT**T**GG**G***A*CG**CCCA**GTCGCTGG | 6/1 | chr8:11318682 -11318705 | *FAM167A* |
| 151 | GC**G**C**CGCC**C*G*CG**T**AGTGTCGCCGG | 6/1 | chr8:61591820 -61591843 | *CHD7* |
| 152 | G**A**T**GG**AG**T**C*T***T**G**C**AGTGTCGCTGG | 6/1 | chr8:98210999-98211022 | Intergenic |
| 153 | G**G**T**TG**AGGC*A***G**GA**G**G**C**GTCGCTGG | 6/1 | chr8:143260245 -143260268 | Intergenic |
| 154 | **AGG**C**GG**GG**G**CGAAGT*G*GTCGCTGG | 6/1 | chr8: 41735234-41735257 | *ANK1* |
| 155 | GCTC**C**AGc**AG**G**C**AG**A***A*GTCGCCGG | 6/1 | chr8:120867894 -120867917 | *DSCC1* |
| 156 | GC**C**C*A***G**A**A**GCC**ATC**GTGTCGCTGG | 6/1 | chr8:12042999-12043022 | *FAM86B1* |
| 157 | GC**C**C*A***G**A**A**GCC**ATC**GTGTCGCTGG | 6/1 | chr8:12285280 -12285303 | *FAM86B2* |
| 158 | **T**CTC*T***G**AGG**TA**G**G**AG**C**GTCGCTGG | 6/1 | chr8:145075476-145075499 | *PARP10* |
| 159 | **C**CTCT**G**GG**G**C**TT***C*AG**G**GTCGCAGG | 6/1 | chr8: 65972428- 65972451 | Intergenic |
| 160 | GCT**GA**A**T**G**AA**GA*T***G**GTGTCGCTGG | 6/1 | chr8:144921513-144921536 | *NRBP2* |
| 161 | GCTCT**GAA**C**A**G*A***T**A**C**TGTCGCTGG | 6/1 | chr8:16533918-16533941 | Intergenic |
| 162 | G**TA**-**A**AGG**AT**G**G**AGTGTCGCAGG | 6/1 | chr8:2323548-2323569 | Intergenic |
| 163 | GC**A**-**GG**GGC**T**GA**G**G**C**GTCGCAGG | 6/1 | chr8:11324041-11324062 | *FAM167A* |
| 164 | GC**G**-T**G**GGC**G**G**CCC**TGTCGCCGG | 6/1 | chr8:48677318-48677339 | Intergenic |
| 165 | **T**CTCT**T**GGCC**C**—GTGTCGCCGG | 3/2 | chr19:37763002-37763022 | Intergenic |
| 166 | **T**CTCT**T**GGCC**C**—GTGTCGCCGG | 3/2 | chr19:37770592-37770612 | Intergenic |
| 167 | GC**A**CT**T**GGC**T**GA—TGTCGCAGG | 3/2 | chr10:71497328 -71497348 | Intergenic |
| 168 | G**A**TCT**G**GGC**A**G—GTGTCGCTGG | 3/2 | chr14:100913107-100913127 | *WDR25* |
| 169 | GCTC**A**AGGCC**CC**—TGTCGCTGG | 3/2 | chrX:70369551-70369571 | *NLGN3* |
| 170 | GCT**GC**AGGC**G**—AGTGTCGCTGG | 3/2 | chr7:102180465-102180485 | *POLR2J3* |
| 171 | GCT**GC**AGGC**G**—AGTGTCGCTGG | 3/2 | chr7:102279573-102279593 | *UPK3BL* |
| 172 | **A**CT**AA**AGGCC—AGTGTCGCTGG | 3/2 | chr21:44988824-44988844 | *HSF2BP* |
| 173 | GCT**G**T**TT**GCC—AGTGTCGCTGG | 3/2 | chr10:125129485-125129505 | Intergenic |
| 174 | GC**C**CT—**G**CCGAAG**G**GTCGCAGG | 3/2 | chr2:197183519 -197183539 | *HECW2* |
| 175 | GC**G**C—GGC**T**G**C**AGTGTCGCAGG | 3/2 | chr7:34360998-34361018 | Intergenic |
| 176 | **AG**TCTAGG—GA**T**GTGTCGCTGG | 3/2 | chr4:24654534-24654554 | Intergenic |
| 177 | GC**A**C—GG**G**CGAAG**C**GTCGCGGG | 3/2 | chr5:93954697-93954717 | *SLF1* |
| 178 | GCTCTAGGC**T**G**T**—**G**GTCGCTGG | 3/2 | chr16:4984707-4984727 | *PPL* |
| 179 | GCT**G**—GGCC**C**AAG**G**GTCGCAGG | 3/2 | chr2: 232051272-232051292 | Intergenic |
| 180 | G**G**TCTAGGCC**T**—G**G**GTCGCGGG | 3/2 | chr2:74742033-74742053 | *TLX2* |
| 181 | GCTC—GGCC**TCG**GTGTCGCGGG | 3/2 | chr19:56186727-56186747 | *EPN1* |
| 182 | GCTC**C**AGG**G**C—AG**A**GTCGCAGG | 3/2 | chr15:51427782-51427802 | Intergenic |
| 183 | **CA**TCTAGGCC—AG**G**GTCGCTGG | 3/2 | chr15:78270624-78270644 | Pseudogene |
| 184 | **CA**TCTAGGCC—AG**G**GTCGCTGG | 3/2 | chr15:79067671-9067691 | Intergenic |
| 185 | **C**CTCT**G**GG—GA**G**GTGTCGCTGG | 3/2 | chr20:58033699-58033719 | Intergenic |
| 186 | **C**CTC**G**AGGCC**TG**—TGTCGCTGG | 4/2 | chr3:50388167-50388187 | *NPRL2/CYB561D2* |
| 187 | GCT**G**T**GA**GC**T**GA—TGTCGCAGG | 4/2 | chr3:64388910-64388930 | *PRICKLE2* |
| 188 | **CGG**CTAGGCC**C**—GTGTCGCGGG | 4/2 | chr3:169899326 -169899346 | *PHC3* |
| 189 | GCTCT**G**GG**TTT**A—TGTCGCTGG | 4/2 | chr7: 75610691-75610711 | *POR* |
| 190 | **AAG**C**C**AGGCCGA—TGTCGCAGG | 4/2 | chr7:101566496-101566516 | *CUX1* |
| 191 | cCTCT**GT**G**T**CGA—TGTCGCTGG | 4/2 | chr7: 130487679-130487699 | Intergenic |
| 192 | **AT**T**T**T**G**GGCC—AGTGTCGCTGG | 4/2 | chr7:2678363 -2678383 | *TTYH3* |
| 193 | G**AGGA**AGG—GAAGTGTCGCTGG | 4/2 | chr3:39247858 -39247878 | Intergenic |
| 194 | GCTCT*TC***G**G**C**CC**A**A**C**GTGTCGCTGG | 4/2 | chr4:156298074-156298098 | *MAP9* |
| 195 | GCT**GAG**GGCCG**T***TG*AGTGTCGCAGG | 4/2 | chr19:38755268 -38755292 | *SPINT2* |
| 196 | G**GC**CT**G**GGCCG*C*AAA**C**TGTCGCCGG | 4/2 | chr11:66328772-66328796 | *ACTN3* |
| 197 | G**AA**CT**G**GGC*AA*CGA**G**GTGTCGCTGG | 4/2 | chr11:120708680-120708704 | *GRIK4* |
| 198 | GC**CT**—GGCCG**G**AG**A**GTCGCTGG | 4/2 | chr8:49959176- 49959196 | Intergenic |
| 199 | G**GG**C—G**C**CCG**C**AGTGTCGCTGG | 4/2 | chr8:55379576- 55379596 | Intergenic |
| 200 | G**T**TCT**T**G**A**C**T**GAA—GTCGCAGG | 4/2 | chr8:28184653 -28184673 | *PNOC* |
| 201 | GCTCT—GCC**AGC**G**A**GTCGCTGG | 4/2 | chr8:143396024-143396044 | *TSNARE1* |
| 202 | G**TC**CT**C**GGCC—AG**A**GTCGCAGG | 4/2 | chr12:2761398-2761418 | *CACNA1C* |
| 203 | G—CTA**T**G**AA**G**C**AGTGTCGCAGG | 4/2 | chr12:130501419-130501439 | Intergenic |
| 204 | GC**CT**TAG**C**C—AA**C**TGTCGCTGG | 4/2 | chr12:16306492-16306512 | Intergenic |
| 205 | GC**A**C**C**A—C**A**GA**C**GTGTCGCTGG | 4/2 | chr12:53238462-53238482 | *KRT78* |
| 206 | G**GA**C**A**AGGCCG**G**A—GTCGCAGG | 4/2 | chr3:197476792-197476812 | *FYTTD1* |
| 207 | G**G**T**GG**AGGCCGAAGG*G***A**GTCGCCGG | 4/2 | chr7:1286878-1286902 | Intergenic |
| 208 | GCTC**CCA**GCCGA**G**G*CC*TGTCGCTGG | 4/2 | chr7:73497873-73497897 | *LIMK1* |
| 209 | GCTC**C**AGG**A**CG**G**—**G**GTCGCAGG | 4/2 | chr7:771507-771527 | *DNAAF5* |
| 210 | GCT**G**T**G**GGCC**C**A—**G**GTCGCTGG | 4/2 | chr7:1524267-1524287 | *INTS1* |
| 211 | **TG**TC**C**AGGC**A**GAAG*AC*TGTCGCAGG | 4/2 | chr5:134951790-134951814 | Intergenic |
| 212 | GCTC**C**AG*AC*G**TT**GA**T**GTGTCGCTGG | 4/2 | chr16: 8491998-8492022 | Intergenic |
| 213 | GC*AG***C**CTAG**C**CCGA**G**G**G**GTCGCTGG | 4/2 | chr1:3425566-3425590 | *MEGF6* |
| 214 | GCTC**ACA**GCCG*GC*AA**T**TGTCGCTGG | 4/2 | chr1:116653380-116653404 | Intergenic |
| 215 | G**TG**C**G**AGGCCG*CC*AAG**C**GTCGCGGG | 4/2 | chr1:153935848-153935872 | *SLC39A1* |
| 216 | **C**CTC**A***TC*AGGC**T**G**C**AGTGTCGCAGG | 4/2 | chr1: 207949438-207949462 | *CD46* |
| 217 | GCTCTAGG**G**C*TT***C**A**G**G**G**GTCGCGGG | 4/2 | chr1:79944127-79944151 | Intergenic |
| 218 | GCTC**ACA**GCCG*GG*A**G**GTGTCGCCGG | 4/2 | chr13:45885373-45885397 | Intergenic |
| 219 | GCT**TC**AGG**GT**GAAGTG*AG*TCGCAGG | 4/2 | chr22:42801782-42801806 | *NFAM1* |
| 220 | **C**CTC**C**AG**A**C**A**GAA*AT*GTGTCGCTGG | 4/2 | chr22:48749585-48749609 | Intergenic |
| 221 | G**A**TC**C**AGGC**A**GA**G**G*GC*TGTCGCTGG | 4/2 | chr19:37855487-37855511 | *HKR1* |
| 222 | GCT**GA**A**A**GCCG*GC*A**G**GTGTCGCAGG | 4/2 | chr21:42746587-42746611 | *MX2* |
| 223 | GCTC**C**AGGCC*CT*G**CCT**TGTCGCTGG | 4/2 | chr10:28109077-28109101 | *ARMC4* |
| 224 | **A**CT**G**T**C**GGCCGA**G**GT*AG*GTCGCGGG | 4/2 | chr9:129375716-129375740 | Intergenic |
| 225 | G**G**TC**G***GG*AGGC**G**GAA**A**TGTCGCTGG | 4/2 | chrX:2876387-2876411 | *ARSL* |
| 226 | **C**CTCTA**TT**CC**CC**—TGTCGCGGG | 5/2 | chr8:125035770-125035790 | *FER1L6* |
| 227 | GCTC**CC**GG**G**C**CC**—TGTCGCTGG | 5/2 | chr8:144789064-144789084 | *CCDC166* |
| 228 | G**T**T**T**T**T**G**TT**C—AGTGTCGCGGG | 5/2 | chr8:2915404-2915424 | *CSMD1* |
| 229 | **T**CTC**AT**GGC**T**—AG**G**GTCGCTGG | 5/2 | chr8:6783552-6783572 | *DEFA6* |
| 230 | **CA**TCT**G**GGC**T**—**T**GTGTCGCCGG | 5/2 | chr8:33267923 -33267943 | *FUT10* |
| 231 | GCT**G**T**G**GGC**A**—A**AG**GTCGCAGG | 5/2 | chr8:97463572-97463592 | Intergenic |
| 232 | G**A**T**GA**AGGC**A**—AG**G**GTCGCAGG | 5/2 | chr8:101398753-101398773 | Intergenic |
| 233 | **TGC**C**G**AGGCCGA—**C**GTCGCAGG | 5/2 | chr8:1645326-1645346 | *DLGAP2* |
| 234 | **C**CTCTAGG**AG**G**G**—**G**GTCGCAGG | 5/2 | chr8:129382928 -129382948 | Intergenic |
| 235 | G**G**T**GC**AGGC**T**GA—**G**GTCGCGGG | 5/2 | chr8:144063710 -144063730 | Intergenic |
| 236 | GCT**G**—G**T**CC**CT**AG**A**GTCGCTGG | 5/2 | chr8: 7327846- 7327866 | *DEFB104B* |
| 237 | GCT**G**—G**T**CC**CT**AG**A**GTCGCTGG | 5/2 | chr8:7698728-7698748 | *DEFB104A* |
| 238 | GC**C**C—**A**G**TA**GAAG**C**GTCGCTGG | 5/2 | chr8:10782269 -10782289 | *XKR6* |
| 239 | GCT**T**—**CC**CC**A**AA**T**TGTCGCTGG | 5/2 | chr8:122558191-122558211 | Intergenic |
| 240 | GCT**G**—GG**GAA**AAG**G**GTCGCTGG | 5/2 | chr8: 145543557-145543577 | *DGAT1* |
| 241 | **A**—CTAGGC**T**G**CTA**TGTCGCAGG | 5/2 | chr8:4277374-4277394 | *CSMD1* |
| 242 | G—CTAGGC**A**G**CCTG**GTCGCCGG | 5/2 | chr8:15398159-15398179 | *TUSC3* |
| 243 | G—C**C**AGG**G**C**CT**AG**G**GTCGCGGG | 5/2 | chr8:38089612-38089635 | *DDHD2* |
| 244 | GCTCT**C**GGC**T**C*T***C**A**CC**TGTCGCGGG | 5/2 | chr8:144643584-144643608 | *GSDMD* |
| 245 | **A**C*AT*T**T**T**T**GGC**A**G**C**AGTGTCGCTGG | 5/2 | chr12:16574917-16574941 | Intergenic |
| 246 | G**A***TT*TCT**CC**G**T**CG**C**AGTGTCGCAGG | 5/2 | chr12: 125877329 -125877353 | *TMEM132B* |
| 247 | G**T**TCTAGG*GC*CC**T**A**GAG**GTCGCAGG | 5/2 | chr12:108199401-108199425 | Intergenic |
| 248 | GC*CA*TCT**GT**G**GT**GAAG**C**GTCGCCGG | 5/2 | chr3:38691473-38691497 | Intergenic |
| 249 | **C**CT*GG*C**C**AGGC**T**G**C**AG**C**GTCGCAGG | 5/2 | chr3:100199135-100199159 | Intergenic |
| 250 | **A**C**ATC**AGGCC*AA*GAAG**A**GTCGCTGG | 5/2 | chr3: 45961365-45961389 | *FYCO1* |
| 251 | GC**GGC**AGGCC*TG*G**C**A**C**TGTCGCCGG | 5/2 | chr3:119528980-119529004 | *NR1I2* |
| 252 | **A**CTCT**CA**G*CT*CC**AT**AGTGTCGCTGG | 5/2 | chr3:194578315-194578339 | Intergenic |
| 253 | G**A**T**GACA**GC**A**—AGTGTCGCAGG | 6/2 | chr8:6931491- 6931511 | Intergenic |
| 254 | G**AAAC**AG**C**C**A**—AGTGTCGCGGG | 6/2 | chr8: 9832229-9832249 | Intergenic |
| 255 | GC**GG**T**CTC**CCGA**C**G*GC*TGTCGCGGG | 6/2 | chr8:12809250 -12809274 | *TRMT9B* |
| 256 | G**G**T**TCT**GGCCGA**GT***CC*TGTCGCAGG | 6/2 | chr8:23563158 – 23563182 | *NKX2-6* |
| 257 | G**TC**C**C**AGGCCG**GCT***CC*TGTCGCCGG | 6/2 | chr8:48651125 – 48651149 | *KIAA0146* |
| 258 | **C**CT*TC*C**AGC**GCCG**G**AG**C**GTCGCGGG | 6/2 | chr8:144649443-144649467 | *MROH6* |
| 259 | **ATG**C**AT**GGCC—**C**GTGTCGCAGG | 6/2 | chr8:962068-962088 | *DLGAP2* |
| 260 | **A**C**C**CT**G**G**C**CC—**T**G**G**GTCGCAGG | 6/2 | chr8:22065131 -22065151 | *BMP1* |
| 261 | **TAC**CTAG**A**C**A**—A**C**TGTCGCTGG | 6/2 | chr8:26027023 -26027043 | Intergenic |
| 262 | **ATC**CT**G**GG**G**C—**T**GTGTCGCAGG | 6/2 | chr8:26131719 -26131739 | Intergenic |
| 263 | **AG**TCT**G**GG**G**C—A**TG**GTCGCTGG | 6/2 | chr8:40862566-40862586 | Intergenic |
| 264 | G**TC**C**CT**GG**G**C—A**T**TGTCGCAGG | 6/2 | chr8:49049208 -49049228 | Intergenic |
| 265 | G**TA**C**C**AG**TT**C—AG**G**GTCGCAGG | 6/2 | chr8:55470386 -55470406 | Intergenic |
| 266 | **C**CTC**GGA**GCC—A**TG**GTCGCGGG | 6/2 | chr8:57390733 -57390753 | Intergenic |
| 267 | G**GCTGT**GGCC—A**A**TGTCGCTGG | 6/2 | chr8: 58860638-58860658 | Intergenic |
| 268 | **AGG**CT**GT**GCCG*CT***C**AGTGTCGCTGG | 6/2 | chr8:41186854 -41186878 | Intergenic |
| 269 | **C**CTCT**G**GG**G**C**T***TC*A**G**G**G**GTCGCAGG | 6/2 | chr8:91431814 -91431838 | *LINC00534* |
| 270 | G**G**TC**C**AG**AG**C**T***CC*A**G**GTGTCGCAGG | 6/2 | chr8:144257991-144258015 | Intergenic |
| 271 | G**A**TC**C**AGGC**T**G*CC*A**GAA**GTCGCTGG | 6/2 | chr8:144509065-144509089 | *MAFA* |
| 272 | GC**C**CT*GC*AGG**TG**G**CC**G**G**GTCGCAGG | 6/2 | chr8:102217459-102217483 | *ZNF706* |
| 273 | GCT**TAT**G**C**C**A**G**T**AGTGTCGCAGT | 6/0 | chr12:95838400-95838422 | Intergenic |
