# Supplementary material for: In silico analysis of potential off-target sites to gene editing for Mucopolysaccharidosis type I using the CRISPR/Cas9 system: Implications for population-specific treatments
Source: PLoS One. 2022 Jan 24;17(1):e0262299. doi: 10.1371/journal.pone.0262299 (PMC8786118; doi:10.1371/journal.pone.0262299)
Supplement: S2 Table — The table shows the cleavage probability for each off-target sequence (with identification tag (ID) following Table 1), off-target annotation, CFD, CFD INDEL and CFD FINAL for all possible off-target sequences returned by the predictors and BWA alignment sequence (ID 273). CFD INDEL with a dash sign means no score was provided for sequences without indel. The CFD FINAL with ** and * are according to CFD > = 0.2 and CFD > = 0.023, respectively. (DOCX) [file pone.0262299.s003.docx]

**S2 Table**

| **ID** | **SEQUENCE** | **ANNOTATION** | **CFD** | **CFD INDEL** | **CFD FINAL** |
| --- | --- | --- | --- | --- | --- |
| 0 | GCTCTAGGCCGAAGTGTCGCAGG | *IDUA* | 1.00000 | - | 1.00000 ** |
| 1 | GCTCTGGGCCGAAGTGTCGCAGG | *IDUA* | 0.71429 | - | 0.71429 ** |
| 2 | GCTCTGGGCTGGGGTGTCGCTGG | *MIR366HG* | 0.31665 | - | 0.31665 ** |
| 3 | TCTCTAGGCAGAAGTGATGCTGG | Intergenic | 0.29714 | - | 0.29714 ** |
| 4 | GCTCTAGGCTGAAGTGCTTCTGG | *CCDC26* | 0.28473 | - | 0.28473 ** |
| 5 | CCACTAGGCCAAAGTGTAGCTGG | *RAB11B* | 0.27473 | - | 0.27473 ** |
| 6 | GCTCCAGGAGGAAGTGTCACAGG | *DCAF1* | 0.23810 | - | 0.23810 ** |
| 7 | GTTCTAGGTGGAAGTGTTGCTGG | *DLC1* | 0.19886 | - | 0.19886 * |
| 8 | GGTATCAGCAGAAGTGTCGCTGG | *PPPR2B* | 0.23077 | - | 0.23077 ** |
| 9 | AATCCAGGTTGAAGTGTCGCCGG | Intergenic | 0.53904 | - | 0.53904 ** |
| 10 | CCACCAGGCTGCAGTGTCGCAGG | Intergenic | 0.12637 | - | 0.12637 * |
| 11 | CCACCAGGCTGCAGTGTCGCAGG | *CAMK1D* | 0.12637 | - | 0.12637 * |
| 12 | GCGCCCGGCCCGAGTGTCGCGGG | Intergenic | 0.07035 | - | 0.07035 * |
| 13 | GCTCCACACAGTAGTGTCGCGGG | Intergenic | 0.19861 | - | 0.19861 * |
| 14 | GATGGAGGCCGCTGTGTCGCAGG | *LHX4* | 0.01837 | - | 0.01837 * |
| 15 | AATCCAGGTCGAAGGGTCGCCGG | Intergenic | 0.02864 | - | 0.02864 * |
| 16 | AATCCAGGTCGAAGGGTCGCCGG | Intergenic | 0.02864 | - | 0.02864 * |
| 17 | AATCCAGGTCGAAGGGTCGCTGG | Intergenic | 0.02864 | - | 0.02864 * |
| 18 | AATCCAGGTCGAAGGGTCGCCGG | Intergenic | 0.02864 | - | 0.02864 * |
| 19 | AATCCAGGCTGAAGGGTCGCTGG | Intergenic | 0.03080 | - | 0.03080 * |
| 20 | TTTCTAGGCTCACGTGTCGCTGG | Intergenic | 0.07720 | - | 0.07720 * |
| 21 | GGTCAACCCCGAAGAGTCGCCGG | Intergenic | 0.08520 | - | 0.08520 * |
| 22 | GCCCTGGTCCGCTGTGTCGCTGG | *GNA13* | 0.01510 | - | 0.01510 |
| 23 | GCTCGAGATCAAGGTGTCGCAGG | *SHANK2* | 0.36522 | - | 0.36522 ** |
| 24 | GATCTGGGGAGAAGGGTCGCAGG | *IL4I1* | 0.01394 | - | 0.01394 |
| 25 | CTTCCAGGCCGAGGAGTCGCCGG | *USP20* | 0.24518 | - | 0.24518 ** |
| 26 | GGTCTAGGCCAAGCTGTCGCTGG | *ASIC2* | 0.19444 | - | 0.19444 * |
| 27 | GCGCTGGGCGGGATTGTCGCAGG | *SHH* | 0.02675 | - | 0.02675* |
| 28 | GCTTTGGGCGGCAGGGTCGCAGG | *LINGO3* | 0.00292 | - | 0.00292 |
| 29 | GTTCTTGGGCGATGAGTCGCGGG | *TAPBP* | 0.06982 | - | 0.06982 * |
| 30 | GCTGAAGGCCTGAGGGTCGCCGG | *TM6SF1* | 0.00347 | - | 0.00347 |
| 31 | GCTCTAATCATAAGCGTCGCAGG | *PHKB* | 0.05682 | - | 0.05682 * |
| 32 | GCACTACACCGAAACGTCGCGGG | Intergenic | 0.10045 | - | 0.10045 * |
| 33 | GCTCGAGCCCAGAGGGTCGCCGG | *RICTOR* | 0.01422 | - | 0.01422 |
| 34 | GATCTAGGCTAAGGAGTCGCAGG | *CHCHD6* | 0.25845 | - | 0.25845 ** |
| 35 | GCTCTAAGCTGGCCTGTCGCTGG | Intergenic | 0.06133 | - | 0.06133 * |
| 36 | ACTGTAGTCCTACATGTCGCTGG | Intergenic | 0.01708 | - | 0.01708 |
| 37 | GCTCCTCCAGGAAGTGTCGCAGG | *ENTPD4* | 0.10073 | - | 0.10073 * |
| 38 | ATTTTTGGCAGCAGTGTCGCTGG | Intergenic | 0.10663 | - | 0.10663 * |
| 39 | TCTCTTTGGCAAAGGGTCGCTGG | Intergenic | 0.01263 | - | 0.01263 |
| 40 | TGTCCTTGCTGAAGTGTCGCAGG | *LINC02439* | 0.26724 | - | 0.26724 ** |
| 41 | TTTCTCCGTCGCAGTGTCGCAGG | *TMEM132B* | 0.06542 | - | 0.06542 * |
| 42 | GTGCTGGGCCTATTTGTCGCTGG | Intergenic | 0.00999 | - | 0.00999 |
| 43 | GCTCTTCTGCCAGGTGTCGCTGG | *MYH15* | 0.05310 | - | 0.05310 * |
| 44 | AATCCAGGTTGAAGGGTCGCTGG | *C3orf70* | 0.02695 | - | 0.02695 * |
| 45 | GCCCTAGGCATATCAGTCGCTGG | *LIN02036* | 0.01063 | - | 0.01063 |
| 46 | GCTCCTCTACCAAGTGTCGCAGG | Intergenic | 0.11275 | - | 0.11275 * |
| 47 | GCCCCAAGTAGAAGGGTCGCAGG | Intergenic | 0.01625 | - | 0.01625 |
| 48 | CCTCGTCGCCGCTGTGTCGCTGG | *C7orf50* | 0.01772 | - | 0.01772 |
| 49 | GTTAAAGAACAAAGTGTCGCTGG | *SDK1* | 0.32809 | - | 0.32809 ** |
| 50 | GCCCTCGTCCGTCTTGTCGCAGG | *HOXA7* | 0.00228 | - | 0.00228 |
| 51 | CCTGTTATCAGAAGTGTCGCAGG | *DBNL* | 0.13818 | - | 0.13818 * |
| 52 | GGACTGTGGGGAAGTGTCGCGGG | *VOPP1* | 0.04883 | - | 0.04883 * |
| 53 | CACCCAGGCTGGAGTGTCGCGGG | Intergenic | 0.15133 | - | 0.15133 * |
| 54 | ACTCTGGGCCAGGCTGTCGCAGG | *TMEM178B* | 0.12977 | - | 0.12977 * |
| 55 | GATCAAGGCTACTGTGTCGCTGG | Intergenic | 0.02702 | - | 0.02702 * |
| 56 | ACTACCGGCAGGAGTGTCGCCGG | *ABLIM2* | 0.21563 | - | 0.21563 ** |
| 57 | ACTCTGTGCTCATGTGTCGCAGG | Intergenic | 0.04445 | - | 0.04445 * |
| 58 | ATCCTAGGCCCCTGTGTCGCAGG | *ARJS* | 0.01186 | - | 0.01186 |
| 59 | GCTCTGCCCTGAGGGGTCGCAGG | *CTNND2* | 0.00927 | - | 0.00927 |
| 60 | TGTTCAGGCCTCAGTGTCGCTGG | *GHR* | 0.05633 | - | 0.05633 * |
| 61 | AATCCAGGTTGAAGAGTCGCTGG | Intergenic | 0.31207 | - | 0.31207 ** |
| 62 | ACTGCAGGTGGAAATGTCGCAGG | Intergenic | 0.11484 | - | 0.11484 * |
| 63 | GATGCAAGAGGAAGTGTCGCTGG | *MCTP1* | 0.12121 | - | 0.12121 * |
| 64 | CTTCCGGGCCGCAGGGTCGCGGG | *FBLL1* | 0.00610 | - | 0.00610 |
| 65 | GCCCTCAGGCAAAGAGTCGCAGG | *LINC00534* | 0.06982 | - | 0.06982 * |
| 66 | GCTCCTGGCGGTTGCGTCGCTGG | *ZC3H3* | 0.00758 | - | 0.00758 |
| 67 | GGTTGAGGTGGAAGAGTCGCTGG | *SCRN1* | 0.07017 | - | 0.07017 * |
| 68 | GTCCAAGGCAGGAGGGTCGCTGG | *CPA5* | 0.00610 | - | 0.00610 |
| 69 | GGACCAGGCGGAGGCGTCGCGGG | *CUL1* | 0.03437 | - | 0.03437 * |
| 70 | AATCCAGGTCAAAGGGTCGCTGG | Intergenic | 0.02864 | - | 0.02864 * |
| 71 | AATCCAGGTTGAAGGGTCGCTGG | Intergenic | 0.02695 | - | 0.02695 * |
| 72 | GCTCTCTGACCAACAGTCGCAGG | Pseudogene | 0.02367 | - | 0.02367 * |
| 73 | TTTCTAGCCAGGACTGTCGCTGG | Intergenic | 0.15007 | - | 0.15007 * |
| 74 | GCTAAAAGTCAAGGTGTCGCAGG | Intergenic | 0.24027 | - | 0.24027 ** |
| 75 | GCTC-AGGCTGAAGGGTCGCAGG | *HERC3* | 0.04706 | 0.00000 | 0.00000 |
| 76 | GCTCTGGGACG-AGTGTCGCTGG | Intergenic | 0.61224 | 0.07143 | 0.04373 * |
| 77 | GCTCTG-GCCGAAGTGACTCAGG | Intergenic | 0.25397 | 0.05882 | 0.01494 |
| 78 | ACTCTATGCTGA-GTGTCGCTGG | *CUX2* | 0.48403 | 0.07692 | 0.03723 * |
| 79 | GAACTAGGCCGTA-TGTCGCTGG | Intergenic | 0.17316 | 0.00000 | 0.00000 |
| 80 | GCGCTG-GCCGCAGAGTCGCCGG | Intergenic | 0.05441 | 0.05882 | 0.00320 |
| 81 | ACT-GAGGTCGAAGTGTCGCTGG | Intergenic | 0.50400 | 0.17647 | 0.08894 * |
| 82 | CCCCTAGGCCTAAG-GTCGCGGG | *NINJ1* | 0.11774 | 0.00000 | 0.00000 |
| 83 | GCGCT-GGCCGCAGAGTCGCCGG | Intergenic | 0.07618 | 0.06667 | 0.00508 |
| 84 | GCGCT-GGCCGCAGAGTCGCCGG | Intergenic | 0.07618 | 0.06667 | 0.00508 |
| 85 | GCTTTAGGGCCAAG-GTCGCTGG | Intergenic | 0.21224 | 0.00000 | 0.00000 |
| 86 | GCTCTAGTACAG-GTGTCGCTGG | Intergenic | 0.38690 | 0.07692 | 0.02976 |
| 87 | GATCTATACCCA-GTGTCGCTGG | *POM121C* | 0.17811 | 0.07692 | 0.01370 * |
| 88 | TCTCTTGGGCCA-GTGTCGCAGG | Intergenic | 0.18950 | 0.07692 | 0.01458 |
| 89 | GCAATAGAG-GAAGTGTCGCTGG | Intergenic | 0.37236 | 0.13333 | 0.04965 * |
| 90 | ACTCTGAGGCCAAGGTGTCGCAGG | Intergenic | 0.41925 | 0.32813 | 0.13757 * |
| 91 | GCACGTGGGCCGAGGGGTCGCGGG | Intergenic | 0.01664 | 0.29688 | 0.00494 |
| 92 | GCTTCCGGGCCGCAGGGTCGCGGG | *FBLL1* | 0.00940 | 0.65625 | 0.00617 |
| 93 | GCGCGGGGCCAGAAGGGTCGCGGG | *IFFO2* | 0.01143 | 0.27692 | 0.00316 |
| 94 | GCTATGGGCAGAGGCTGTCGCTGG | Intergenic | 0.33998 | 0.00000 | 0.00000 |
| 95 | GCTCCACGCTGATG-GTCGCTGG | *CLN8* | 0.19412 | 0.00000 | 0.00000 |
| 96 | CCTCTTCACC-AAGTGTCGCAGG | *OR10P1* | 0.35077 | 0.10000 | 0.03508 * |
| 97 | CCTCTAGACC-AGGGGTCGCAGG | Intergenic | 0.02329 | 0.10000 | 0.00233 |
| 98 | GGTCTAGCCTGA-GGGTCGCAGG | Intergenic | 0.02015 | 0.07692 | 0.00155 |
| 99 | GCACGCGGCC-AGGTGTCGCCGG | Intergenic | 0.13552 | 0.10000 | 0.01355 |
| 100 | GCGCTAGGAT-ACGTGTCGCGGG | *MRPS22* | 0.08492 | 0.10000 | 0.00849 |
| 101 | GCTG-AGGCAGGAGAGTCGCTGG | Intergenic | 0.18119 | 0.00000 | 0.00000 |
| 102 | CCTCTAGGCAGGTGTGGTCGCTGG | *DPP6* | 0.13413 | 0.00000 | 0.00000 |
| 103 | GCT-GGGGCAGAGGTGTCGCTGG | Intergenic | 0.25839 | 0.17647 | 0.04560 * |
| 104 | GCT-GAGGCAGGAGAGTCGCTGG | *PMS2* | 0.23192 | 0.17647 | 0.04093 * |
| 105 | GCT-GAGGCACAAGAGTCGCTGG | Intergenic | 0.13762 | 0.17647 | 0.02429 * |
| 106 | GCTCGAGGCC-GTGCGTCGCGGG | Intergenic | 0.03782 | 0.10000 | 0.00378 |
| 107 | GCT-GAGGCAGGAGAGTCGCTGG | Intergenic | 0.23192 | 0.17647 | 0.04093 * |
| 108 | GCT-GAGGCAGGAGAGTCGCTGG | *GALNTL6* | 0.23192 | 0.17647 | 0.04093 * |
| 109 | ACTCTATGCC-AGGCGTCGCTGG | *TENM3-AS1* | 0.09147 | 0.10000 | 0.00915 |
| 110 | GCT-TTGGCAGGAGGGTCGCTGG | *ADCY2* | 0.02235 | 0.17647 | 0.00394 |
| 111 | GAGCTAGGCCGATGGTGTCGCTGG | *SNX29* | 0.00545 | 0.00000 | 0.00000 |
| 112 | GCAGTAGGCCCGAGTCGTCGCCGG | *CRNDE* | 0.11054 | 0.00000 | 0.00000 |
| 113 | GCTA-AGGCAGGAGGGTCGCTGG | *GCSH* | 0.02635 | 0.00000 | 0.00000 |
| 114 | GCTC-AGCCCTAGCTGTCGCTGG | *PIEZO1* | 0.06615 | 0.00000 | 0.00000 |
| 115 | GCTCT-GGGGGATGAGTCGCAGG | *CDH13* | 0.04181 | 0.06667 | 0.00279 |
| 116 | GCTCTCAGACCATGGTGTCGCTGG | *ANKRD11* | 0.21739 | 0.43750 | 0.09511 * |
| 117 | GCTGCAGACACGATGTGTCGCGGG | Intergenic | 0.15000 | 0.56923 | 0.08538 * |
| 118 | GCTCCAGGCTCGGCGGGTCGCGGG | *MAPKAPK2* | 0.00760 | 0.65079 | 0.00495 |
| 119 | GCT-CAGGCAGTAATGTCGCCGG | *USH2A* | 0.21667 | 0.17647 | 0.03824 * |
| 120 | GCT-GAGGCAGGAGAGTCGCTGG | *PCNX2* | 0.23192 | 0.17647 | 0.04093 * |
| 121 | GCTGGA-GCCGAGGCGTCGCAGG | *CNIH3* | 0.05692 | 0.05882 | 0.00335 |
| 122 | CCTCCGGGCCGAGG-GTCGCGGG | Intergenic | 0.33274 | 0.00000 | 0.00000 |
| 123 | GTTCTTAGTCCAATGTGTCGCCGG | *SH3BP4* | 0.17045 | 0.21538 | 0.03671 * |
| 124 | GCTTTTGATCGTAAGTGTCGCTGG | *R3HDM4* | 0.50000 | 0.03077 | 0.01538 |
| 125 | GCTCCAGGAGAGAAGGGTCGCTGG | *RAP1GAP2* | 0.01667 | 0.27692 | 0.00462 |
| 126 | GGTCTCAGGCGCAGGTGTCGCGGG | *PAX2* | 0.07561 | 0.43750 | 0.03308 * |
| 127 | GCTCTACAGCAGGAGGGTCGCGGG | *DPF3* | 0.03130 | 0.56923 | 0.01781 |
| 128 | GCGTGAAGGCGGCAGTGTCGCCGG | *AOPEP* | 0.02558 | 0.82258 | 0.02105 |
| 129 | CCCCTAAGCTGCAAGTGTCGCTGG | Intergenic | 0.28812 | 0.04688 | 0.01351 |
| 130 | CCCCTAAGCTGCAAGTGTCGCTGG | Intergenic | 0.28812 | 0.04688 | 0.01351 |
| 131 | TCTCCAGGCCCCCAGCGTCGCAGG | Intergenic | 0.03076 | 0.41538 | 0.01278 |
| 132 | GCTGCGGACTCCAAGTGTCGCCGG | *LNCOC1* | 0.15306 | 0.65079 | 0.09961 * |
| 133 | GGT-GAGGGAGGAGTGTCGCTGG | *PEBP4* | 0.17251 | 0.17647 | 0.03044 * |
| 134 | GCT-GAGGGAGACTTGTCGCAGG | Intergenic | 0.01928 | 0.17647 | 0.00340 |
| 135 | GCC-TGGGCAGCATTGTCGCAGG | *GPIHBP1* | 0.01862 | 0.17647 | 0.00329 |
| 136 | ACTCTGAGGCCAAGGTGTCGCAGG | Intergenic | 0.17055 | 0.06349 | 0.01083 |
| 137 | GCCCCTGGCC-ACCTGTCGCGGG | *TSNARE1* | 0.02762 | 0.10000 | 0.00276 |
| 138 | GGTC-AGGTTTAAGGGTCGCTGG | *MCPH-AS1* | 0.01102 | 0.00000 | 0.00000 |
| 139 | GC-CAAGGTGGGAGGGTCGCTGG | *PLAT* | 0.00614 | 0.60000 | 0.00369 |
| 140 | GA-CTTGGCTGCAGAGTCGCAGG | *LOC102724708* | 0.07449 | 0.60000 | 0.04469 * |
| 141 | CATCAA-GCTGCAGTGTCGCAGG | Intergenic | 0.06433 | 0.05882 | 0.00378 |
| 142 | GCTCT-GTTCGTGCTGTCGCTGG | *MYOM2* | 0.05095 | 0.06667 | 0.00340 |
| 143 | GCTGCAGGCGGGAGGGGTCGCGGG | *DCNT2* | 0.00702 | 0.00000 | 0.00000 |
| 144 | GGTTCAGACCCAAGTAGTCGCAGG | Intergenic | 0.23851 | 0.01538 | 0.00367 |
| 145 | GCGCATAGCCGTAGTCGTCGCAGG | *SCAP* | 0.05952 | 0.00000 | 0.00000 |
| 146 | GCTCATAAGCAGGGCTGTCGCTGG | Intergenic | 0.00000 | 0.00000 | 0.00000 |
| 147 | GATTCAGGTCCGCAGGGTCGCTGG | *STIMATE-MUSTN1* | 0.00766 | 0.60938 | 0.00467 |
| 148 | GGTCTACACCCACAGTGTCGCTGG | *POM121* | 0.12586 | 0.61538 | 0.07745 * |
| 149 | GGTCTACACCCACAGTGTCGCTGG | *POM121B* | 0.12586 | 0.61538 | 0.07745 * |
| 150 | GCTCTTGGGACGCCCAGTCGCTGG | *FAM167A* | 0.00608 | 0.56923 | 0.00346 |
| 151 | GCGCCGCCCGCGTAGTGTCGCCGG | *CHD7* | 0.05037 | 0.58730 | 0.02958 * |
| 152 | GATGGAGTCTTGCAGTGTCGCTGG | *LOC101927066* | 0.03603 | 0.65079 | 0.02345 * |
| 153 | GGTTGAGGCAGGAGGCGTCGCTGG | Intergenic | 0.02464 | 0.56923 | 0.01402 |
| 154 | AGGCGGGGGCGAAGTGGTCGCTGG | *ANK1* | 0.08859 | 0.00000 | 0.00000 |
| 155 | GCTCCAGCAGGCAGAAGTCGCCGG | *DSCC1* | 0.03125 | 0.01538 | 0.00048 |
| 156 | GCCCAGAAGCCATCGTGTCGCTGG | *FAM86B1* | 0.01925 | 0.32813 | 0.00632 |
| 157 | GCCCAGAAGCCATCGTGTCGCTGG | *FAM86B1* | 0.01925 | 0.32813 | 0.00632 |
| 158 | TCTCTGAGGTAGGAGCGTCGCTGG | Intergenic | 0.09560 | 0.21538 | 0.02059 |
| 159 | CCTCTGGGGCTTCAGGGTCGCAGG | Intergenic | 0.00202 | 0.00000 | 0.00000 |
| 160 | GCTGAATGAAGATGGTGTCGCTGG | *NRBP2* | 0.06921 | 0.00000 | 0.00000 |
| 161 | GCTCTGAACAGATACTGTCGCTGG | Intergenic | 0.08844 | 0.06154 | 0.00544 |
| 162 | GTA-AAGGATGGAGTGTCGCAGG | Intergenic | 0.18917 | 0.17647 | 0.03338 * |
| 163 | GCA-GGGGCTGAGGCGTCGCAGG | *FAM167A* | 0.05466 | 0.17647 | 0.00965 |
| 164 | GCG-TGGGCGGCCCTGTCGCCGG | Intergenic | 0.00330 | 0.17647 | 0.00058 |
| 165 | TCTCTTGGCCC--GTGTCGCCGG | Intergenic | 0.30612 | 0.00549 | 0.00168 |
| 166 | TCTCTTGGCCC--GTGTCGCCGG | Intergenic | 0.30612 | 0.00549 | 0.00168 |
| 167 | GCACTTGGCTGA--TGTCGCAGG | Intergenic | 0.48019 | 0.00000 | 0.00000 |
| 168 | GATCTGGGCAG--GTGTCGCTGG | *WDR25* | 0.45022 | 0.00549 | 0.00247 |
| 169 | GCTCAAGGCCCC--TGTCGCTGG | *NLGN3* | 0.05639 | 0.00000 | 0.00000 |
| 170 | GCTGCAGGCG--AGTGTCGCTGG | *POLR2J3* | 0.19444 | 0.00714 | 0.00139 |
| 171 | GCTGCAGGCG--AGTGTCGCTGG | *UPK3BL* | 0.19444 | 0.00714 | 0.00139 |
| 172 | ACTAAAGGCC--AGTGTCGCTGG | *HSF2BP* | 0.37895 | 0.00714 | 0.00271 |
| 173 | GCTGTTTGCC--AGTGTCGCTGG | Intergenic | 0.20408 | 0.00714 | 0.00146 |
| 174 | GCCCT--GCCGAAGGGTCGCAGG | *HECW2* | 0.02143 | 0.00392 | 0.00008 |
| 175 | GCGC--GGCTGCAGTGTCGCAGG | Intergenic | 0.12384 | 0.00000 | 0.00000 |
| 176 | AGTCTAGG--GATGTGTCGCTGG | Intergenic | 0.18783 | 0.00000 | 0.00000 |
| 177 | GCAC--GGGCGAAGCGTCGCGGG | *SLF1* | 0.12059 | 0.00000 | 0.00000 |
| 178 | GCTCTAGGCTGT--GGTCGCTGG | *PPL* | 0.01569 | 0.00000 | 0.00000 |
| 179 | GCTG--GGCCCAAGGGTCGCAGG | Intergenic | 0.01071 | 0.00000 | 0.00000 |
| 180 | GGTCTAGGCCT--GGGTCGCGGG | *TLX2* | 0.01338 | 0.00549 | 0.00007 |
| 181 | GCTC--GGCCTCGGTGTCGCGGG | *EPN1* | 0.06601 | 0.00000 | 0.00000 |
| 182 | GCTCCAGGGC--AGAGTCGCAGG | *MIR4713HG* | 0.35840 | 0.00714 | 0.00256 |
| 183 | CATCTAGGCC--AGGGTCGCTGG | Pseudogene | 0.02597 | 0.00714 | 0.00019 |
| 184 | CATCTAGGCC--AGGGTCGCTGG | *ADAMTS7* | 0.02597 | 0.00714 | 0.00019 |
| 185 | CCTCTGGG--GAGGTGTCGCTGG | Intergenic | 0.33274 | 0.00000 | 0.00000 |
| 186 | CCTCGAGGCCTG--TGTCGCTGG | *NPRL2/CYB561D2* | 0.12698 | 0.00000 | 0.00000 |
| 187 | GCTGTGAGCTGA--TGTCGCAGG | Intergenic | 0.33613 | 0.00000 | 0.00000 |
| 188 | CGGCTAGGCCC--GTGTCGCGGG | *PHC3* | 0.10648 | 0.00549 | 0.00059 |
| 189 | GCTCTGGGTTTA--TGTCGCTGG | *POR* | 0.22624 | 0.00000 | 0.00000 |
| 190 | AAGCCAGGCCGA--TGTCGCAGG | *CUX1* | 0.32727 | 0.00000 | 0.00000 |
| 191 | CCTCTGTGTCGA--TGTCGCTGG | Intergenic | 0.25510 | 0.00000 | 0.00000 |
| 192 | ATTTTGGGCC--AGTGTCGCTGG | *TTYH3* | 0.46753 | 0.00714 | 0.00334 |
| 193 | GAGGAAGG--GAAGTGTCGCTGG | Intergenic | 0.09091 | 0.00000 | 0.00000 |
| 194 | GCTCTTCGGCCCAACGTGTCGCTGG | *MAP9* | 0.09254 | 0.19519 | 0.01806 |
| 195 | GCTGAGGGCCGTTGAGTGTCGCAGG | *SPINT2* | 0.12897 | 0.00095 | 0.00012 |
| 196 | GGCCTGGGCCGCAAACTGTCGCCGG | *ACTN3* | 0.09127 | 0.00288 | 0.00026 |
| 197 | GAACTGGGCAACGAGGTGTCGCTGG | *GRIK4* | 0.24199 | 0.32402 | 0.07841 * |
| 198 | GCCT--GGCCGGAGAGTCGCTGG | Intergenic | 0.14336 | 0.00000 | 0.00000 |
| 199 | GGGC--GCCCGCAGTGTCGCTGG | Intergenic | 0.05633 | 0.00000 | 0.00000 |
| 200 | GTTCTTGACTGAA--GTCGCAGG | *PNOC* | 0.61115 | 0.00000 | 0.00000 |
| 201 | GCTCT--GCCAGCGAGTCGCTGG | *TSNARE1* | 0.08803 | 0.00392 | 0.00035 |
| 202 | GTCCTCGGCC--AGAGTCGCAGG | *CACNA1C* | 0.10253 | 0.00714 | 0.00073 |
| 203 | G--CTATGAAGCAGTGTCGCAGG | Intergenic | 0.11171 | 0.42857 | 0.04787 * |
| 204 | GCCTTAGCC--AACTGTCGCTGG | Intergenic | 0.09042 | 0.01333 | 0.00121 |
| 205 | GCACCA--CAGACGTGTCGCTGG | *KRT78* | 0.13033 | 0.00784 | 0.00102 |
| 206 | GGACAAGGCCGGA--GTCGCAGG | *FYTTD1* | 0.17943 | 0.00000 | 0.00000 |
| 207 | GGTGGAGGCCGAAGGGAGTCGCCGG | Intergenic | 0.12888 | 0.00000 | 0.00000 |
| 208 | GCTCCCAGCCGAGGCCTGTCGCTGG | Intergenic | 0.29644 | 0.00000 | 0.00000 |
| 209 | GCTCCAGGACGG--GGTCGCAGG | *DNAAF5* | 0.03095 | 0.00000 | 0.00000 |
| 210 | GCTGTGGGCCCA--GGTCGCTGG | *INTS1* | 0.00765 | 0.00000 | 0.00000 |
| 211 | TGTCCAGGCAGAAGACTGTCGCAGG | *SLC25A48* | 0.60290 | 0.00000 | 0.00000 |
| 212 | GCTCCAGACGTTGATGTGTCGCTGG | Intergenic | 0.24706 | 0.02885 | 0.00713 |
| 213 | GCAGCCTAGCCCGAGGGGTCGCTGG | *MEGF6* | 0.00860 | 0.73118 | 0.00629 |
| 214 | GCTCACAGCCGGCAATTGTCGCTGG | Intergenic | 0.06061 | 0.00586 | 0.00036 |
| 215 | GTGCGAGGCCGCCAAGCGTCGCGGG | *SLC39A1* | 0.07934 | 0.00220 | 0.00017 |
| 216 | CCTCATCAGGCTGCAGTGTCGCAGG | *CD46* | 0.17691 | 0.07067 | 0.01250 |
| 217 | GCTCTAGGGCTTCAGGGGTCGCGGG | Intergenic | 0.00865 | 0.10438 | 0.00090 |
| 218 | GCTCACAGCCGGGAGGTGTCGCCGG | Intergenic | 0.14822 | 0.01563 | 0.00232 |
| 219 | GCTTCAGGGTGAAGTGAGTCGCAGG | *NFAM1* | 0.46611 | 0.00000 | 0.00000 |
| 220 | CCTCCAGACAGAAATGTGTCGCTGG | Intergenic | 0.61905 | 0.00284 | 0.00176 |
| 221 | GATCCAGGCAGAGGGCTGTCGCTGG | *HKR1* | 0.41107 | 0.00000 | 0.00000 |
| 222 | GCTGAAAGCCGGCAGGTGTCGCAGG | *MX2* | 0.16304 | 0.00586 | 0.00096 |
| 223 | GCTCCAGGCCCTGCCTTGTCGCTGG | *ARMC4* | 0.01477 | 0.05467 | 0.00081 |
| 224 | ACTGTCGGCCGAGGTAGGTCGCGGG | Intergenic | 0.13340 | 0.00000 | 0.00000 |
| 225 | GGTCGGGAGGCGGAAATGTCGCTGG | *ARSL* | 0.12986 | 0.15259 | 0.01981 |
| 226 | CCTCTATTCCCC--TGTCGCGGG | *FER1L6* | 0.02877 | 0.00000 | 0.00000 |
| 227 | GCTCCCGGGCCC--TGTCGCTGG | *CCDC166* | 0.03174 | 0.00000 | 0.00000 |
| 228 | GTTTTTGTTC--AGTGTCGCGGG | *CSMD1* | 0.28409 | 0.00714 | 0.00203 |
| 229 | TCTCATGGCT--AGGGTCGCTGG | *DEFA6* | 0.01681 | 0.00714 | 0.00012 |
| 230 | CATCTGGGCT--TGTGTCGCCGG | *FUT10* | 0.10477 | 0.00714 | 0.00075 |
| 231 | GCTGTGGGCA--AAGGTCGCAGG | Intergenic | 0.01161 | 0.00714 | 0.00008 |
| 232 | GATGAAGGCA--AGGGTCGCAGG | Intergenic | 0.00788 | 0.00714 | 0.00006 |
| 233 | TGCCGAGGCCGA--CGTCGCAGG | *DLGAP2* | 0.05204 | 0.00000 | 0.00000 |
| 234 | CCTCTAGGAGGG--GGTCGCAGG | Intergenic | 0.00860 | 0.00000 | 0.00000 |
| 235 | GGTGCAGGCTGA--GGTCGCGGG | *LY6E-DT* | 0.01637 | 0.00000 | 0.00000 |
| 236 | GCTG--GTCCCTAGAGTCGCTGG | *DEFB104B* | 0.02585 | 0.00000 | 0.00000 |
| 237 | GCTG--GTCCCTAGAGTCGCTGG | *DEFB104A* | 0.02585 | 0.00000 | 0.00000 |
| 238 | GCCC--AGTAGAAGCGTCGCTGG | *XKR6* | 0.08864 | 0.00000 | 0.00000 |
| 239 | GCTT--CCCCAAATTGTCGCTGG | Intergenic | 0.09026 | 0.00000 | 0.00000 |
| 240 | GCTG--GGGAAAAGGGTCGCTGG | *DGAT1* | 0.01341 | 0.00000 | 0.00000 |
| 241 | A--CTAGGCTGCTATGTCGCAGG | *CSMD1* | 0.05015 | 0.42857 | 0.02149 |
| 242 | G--CTAGGCAGCCTGGTCGCCGG | *TUSC3* | 0.00064 | 0.42857 | 0.00027 |
| 243 | G--CCAGGGCCTAGGGTCGCGGG | *DDHD2* | 0.00442 | 0.42857 | 0.00190 |
| 244 | GCTCTCGGCTCTCACCTGTCGCGGG | *GSDMD* | 0.01654 | 0.05467 | 0.00090 |
| 245 | ACATTTTTGGCAGCAGTGTCGCTGG | Intergenic | 0.11729 | 0.81944 | 0.09612 * |
| 246 | GATTTCTCCGTCGCAGTGTCGCAGG | *TMEM132B* | 0.05233 | 0.84985 | 0.04447 * |
| 247 | GTTCTAGGGCCCTAGAGGTCGCAGG | Intergenic | 0.00855 | 0.13630 | 0.00117 |
| 248 | GCCATCTGTGGTGAAGCGTCGCCGG | Intergenic | 0.06486 | 0.82963 | 0.05381 * |
| 249 | CCTGGCCAGGCTGCAGCGTCGCAGG | Intergenic | 0.04825 | 0.27361 | 0.01320 |
| 250 | ACATCAGGCCAAGAAGAGTCGCTGG | *FYCO1* | 0.29774 | 0.07669 | 0.02283 |
| 251 | GCGGCAGGCCTGGCACTGTCGCCGG | *NR1I2* | 0.02820 | 0.08077 | 0.00228 |
| 252 | ACTCTCAGCTCCATAGTGTCGCTGG | Intergenic | 0.13636 | 0.25313 | 0.03452 * |
| 253 | GATGACAGCA--AGTGTCGCAGG | Intergenic | 0.07163 | 0.00714 | 0.00051 |
| 254 | GAAACAGCCA--AGTGTCGCGGG | Intergenic | 0.23331 | 0.00714 | 0.00167 |
| 255 | GCGGTCTCCCGACGGCTGTCGCGGG | *TRMT9B* | 0.00841 | 0.00000 | 0.00000 |
| 256 | GGTTCTGGCCGAGTCCTGTCGCAGG | *NKX2-6* | 0.06913 | 0.00000 | 0.00000 |
| 257 | GTCCCAGGCCGGCTCCTGTCGCCGG | *KIAA0146* | 0.01580 | 0.00000 | 0.00000 |
| 258 | CCTTCCAGCGCCGGAGCGTCGCGGG | *MROH6* | 0.03455 | 0.44792 | 0.01547 |
| 259 | ATGCATGGCC--CGTGTCGCAGG | *DLGAP2* | 0.03076 | 0.00714 | 0.00022 |
| 260 | ACCCTGGCCC--TGGGTCGCAGG | *BMP1* | 0.00254 | 0.00714 | 0.00002 |
| 261 | TACCTAGACA--ACTGTCGCTGG | Intergenic | 0.11577 | 0.00714 | 0.00083 |
| 262 | ATCCTGGGGC--TGTGTCGCAGG | Intergenic | 0.04651 | 0.00714 | 0.00033 |
| 263 | AGTCTGGGGC--ATGGTCGCTGG | Intergenic | 0.00369 | 0.00714 | 0.00003 |
| 264 | GTCCCTGGGC--ATTGTCGCAGG | Intergenic | 0.04594 | 0.00714 | 0.00033 |
| 265 | GTACCAGTTC--AGGGTCGCAGG | Intergenic | 0.01776 | 0.00714 | 0.00013 |
| 266 | CCTCGGAGCC--ATGGTCGCGGG | *LOC101929415* | 0.00435 | 0.00714 | 0.00003 |
| 267 | GGCTGTGGCC--AATGTCGCTGG | Intergenic | 0.08177 | 0.00714 | 0.00058 |
| 268 | AGGCTGTGCCGCTCAGTGTCGCTGG | Intergenic | 0.03362 | 0.00144 | 0.00005 |
| 269 | CCTCTGGGGCTTCAGGGGTCGCAGG | Intergenic | 0.00396 | 0.00144 | 0.00001 |
| 270 | GGTCCAGAGCTCCAGGTGTCGCAGG | Intergenic | 0.10802 | 0.00220 | 0.00024 |
| 271 | GATCCAGGCTGCCAGAAGTCGCTGG | Intergenic | 0.19383 | 0.00220 | 0.00043 |
| 272 | GCCCTGCAGGTGGCCGGGTCGCAGG | *ZNF706* | 0.00040 | 0.17090 | 0.00007 |
| 273 | GCTTATGCCAGTAGTGTCGCAGT | Intergenic | 0.00082 | - | 0.00082 |
