# Supplementary material for: In silico analysis of potential off-target sites to gene editing for Mucopolysaccharidosis type I using the CRISPR/Cas9 system: Implications for population-specific treatments
Source: PLoS One. 2022 Jan 24;17(1):e0262299. doi: 10.1371/journal.pone.0262299 (PMC8786118; doi:10.1371/journal.pone.0262299)
Supplement: S3 Table — The table shows sequence identification tag (ID), sequence, genomic localization and the repeat element where signal (-) and signal (*) denote no repeat element found or sequences close to repeat elements in the region evaluated, respectively. (DOCX) [file pone.0262299.s004.docx]

**S3 Table**

| **ID** | **SEQUENCE** | **GENOME LOCALIZATION** | **REPEAT ELEMENT** |
| --- | --- | --- | --- |
| 15 | AATCCAGGTCGAAGGGTCGCCGG | chr16:3131030-3131052 | ERV_classII |
| 16 | AATCCAGGTCGAAGGGTCGCCGG | chr10:81735284-81735306 | ERV_classII |
| 17 | AATCCAGGTCGAAGGGTCGCTGG | chr11:59691555-59691577 | ERV_classII |
| 18 | AATCCAGGTCGAAGGGTCGCCGG | chr6:67041123-67041145 | LINE1/ERV_classII |
| 19 | AATCCAGGCTGAAGGGTCGCTGG | chrY:21455475-21455497 | ERV_classII |
| 83 | GCGCT-GGCCGCAGAGTCGCCGG | chrX:153618565-153618586 | - |
| 84 | GCGCT-GGCCGCAGAGTCGCCGG | chrX:153570256-153570277 | - |
| 90 | ACTCTGAGGCCAAGGTGTCGCAGG | chr8:105318003-105318026 | ERVL-MaLRs |
| 104 | GCT-GAGGCAGGAGAGTCGCTGG | chr7:6037811-6037832 | ALU |
| 107 | GCT-GAGGCAGGAGAGTCGCTGG | chr4:38211033-38211054 | ALU |
| 108 | GCT-GAGGCAGGAGAGTCGCTGG | chr4:173419240-173419261 | ALU |
| 120 | GCT-GAGGCAGGAGAGTCGCTGG | chr1:233151337-233151358 | ALU |
| 129 | CCCCTAAGCTGCAAGTGTCGCTGG | chrX:118942357-118942379 | LINE2 |
| 130 | CCCCTAAGCTGCAAGTGTCGCTGG | chrX:118950255-118950278 | LINE2 |
| 136 | ACTCTGAGGCCAAGGTGTCGCAGG | chr8:105318003-105318026 | LINE2 |
| 148 | GGTCTACACCCACAGTGTCGCTGG | chr7:72415099-72415122 | ERVL-MaLRs |
| 149 | GGTCTACACCCACAGTGTCGCTGG | chr7:72713486-72713509 | LINE2 |
| 156 | GCCCAGAAGCCATCGTGTCGCTGG | chr8:12042999-12043022 | - |
| 157 | GCCCAGAAGCCATCGTGTCGCTGG | chr8:12285280-12285303 | - |
| 165 | TCTCTTGGCCC—GTGTCGCCGG | chr19:37763002-37763022 | SATELITE |
| 166 | TCTCTTGGCCC—GTGTCGCCGG | chr19:37770592-37770612 | SATELITE |
| 170 | GCTGCAGGCG—AGTGTCGCTGG | chr7:102180465-102180485 | MIR* |
| 171 | GCTGCAGGCG—AGTGTCGCTGG | chr7:102279573-102279593 | MIR* |
| 183 | CATCTAGGCC—AGGGTCGCTGG | chr15:78270624-78270644 | MIR* |
| 184 | CATCTAGGCC—AGGGTCGCTGG | chr15:79067671-79067691 | MIR* |
| 236 | GCTG—GTCCCTAGAGTCGCTGG | chr8:7327846-7327866 | - |
| 237 | GCTG—GTCCCTAGAGTCGCTGG | chr8:7698728-7698748 | - |
