# Supplementary material for: In silico analysis of potential off-target sites to gene editing for Mucopolysaccharidosis type I using the CRISPR/Cas9 system: Implications for population-specific treatments
Source: PLoS One. 2022 Jan 24;17(1):e0262299. doi: 10.1371/journal.pone.0262299 (PMC8786118; doi:10.1371/journal.pone.0262299)
Supplement: S4 Table — Allele frequency is shown for three populations (Europe–E, Latin America–LA, Africa–A) and worldwide frequency (–W) according to the 1000 Genomes database. Position of the alternative allele in bold. (DOCX) [file pone.0262299.s005.docx]

**S4 Table**

| **ID** | **SEQUENCE** | **REF** | **ALT** | **E** | **LA** | **A** | **W** |
| --- | --- | --- | --- | --- | --- | --- | --- |
| 5 | CCACTAGGCCAAAGT**GTA**GCTGG | TAC | T | 4.3 | 3.7 | 11.3 | 7.0 |
| 9 | AATCCAGGTTGAAGTGTCGCC**G**G | C | T | 22.3 | 17.7 | 2.7 | 11,6 |
| 10 | CCACCAGGCTGCAGTGTC**G**CAGG | G | A | 25.1 | 24.8 | 12.2 | 18.3 |
| 11 | GGTCTGGGTGTAAGTGTC**G**CGGG | C | T | 0.1 | 0.4 | 4.3 | 1.2 |
| 11 | GGTCTGGGTGTAAGTGT**C**GCGGG | G | A | 0 | 0 | 2.8 | 0.7 |
| 12 | GCGCCCG**G**CCCGAGTGTCGCGGG | C | G | 0 | 0 | 1.4 | 0.4 |
| 16 | AATCCAGGTCGAAGGGTC**G**CCGG | C | T | 7.1 | 15.1 | 1.3 | 8.4 |
| 18 | AATCCAGGTCGAAGGGTCGCC**G**G | C | T | 0.1 | 0.4 | 6.1 | 1.7 |
| 22 | GCCCTGGTC**C**GCTGTGTCGCTGG | G | T | 90.5 | 90.5 | 33.5 | 77.2 |
| 30 | GCTGAAGGCCTGAGGGTCGC**C**GG | C | T | 0.1 | 0 | 2.2 | 0.6 |
| 34 | GATCTAGGCTAAGGAGTC**G**CAGG | C | T | 0 | 0.6 | 4.6 | 1.3 |
| 37 | **GCTCCTCCAGGAAGTGTCGCAGG** | GCTCCT | G | 0 | 0.1 | 1.2 | 0.4 |
| 37 | GCTCCTC**CAGGA**AGTGTCGCAGG | CAGGA | C | 0 | 0.1 | 1.2 | 0.4 |
| 40 | TGTCCTTGCTGAAGTGTC**G**CAGG | G | C | 0 | 0.1 | 1.4 | 0.4 |
| 44 | AATCCAGG**T**TGAAGGGTCGCTGG | T | C | 20.2 | 28.2 | 38.1 | 30.9 |
| 45 | GCCCTA**GGCA**TATCAGTCGCTGG | GGCA | G | 0.8 | 1.0 | 9.1 | 4.1 |
| 48 | CCTCGTCGCCGCTGTGTCG**C**TGG | G | C | 0.1 | 0.6 | 7.7 | 2.1 |
| 48 | CCTC**G**TCGCCGCTGTGTCGCTGG | C | G | 0 | 0.1 | 2.5 | 0.7 |
| 50 | GCCCTCGTCCGTC**T**TGTCGCAGG | T | C | 0.5 | 0.9 | 3.1 | 1.4 |
| 57 | ACTCTGTGCTCATGTGTC**G**CAGG | C | T | 0.3 | 3.0 | 12.8 | 5.2 |
| 58 | ATCCTAGGCCCCTGTGTC**G**CAGG | C | T | 31.0 | 29.0 | 17.1 | 25.4 |
| 59 | GCTCTGCCCTGAGGGGTCGCAG**G** | C | A | 0 | 1.0 | 9.2 | 2.6 |
| 61 | AATCCAGGTTGAAGAGTC**G**CTGG | G | A | 0 | 0 | 1.4 | 0.4 |
| 69 | GGA**C**CAGGCGGAGGCGTCGCGGG | C | T | 0.2 | 0.6 | 10.3 | 2.8 |
| 70 | AATCCAGGTCAAA**G**GGTCGCTGG | G | A | 0 | 0.1 | 1.5 | 0.4 |
| 71 | AATCCAGGTTGAAGGGTC**G**CTGG | C | T | 0 | 0 | 1.4 | 0.4 |
| 73 | T**T**TCTAGCCAGGACTGTCGCTGG | A | G | 0 | 0.1 | 3.0 | 0.8 |
| 75 | GCTC-AGGCTGAAGGGT**C**GCAGG | C | T | 0 | 0,6 | 3.1 | 0.9 |
| 75 | GCTC-AGGCTGAA**G**GGTCGCAGG | G | C | 0 | 0 | 1.4 | 0.4 |
| 82 | CCCCTAGGCCTAAG-GTCG**C**GGG | C | T | 81.0 | 87.8 | 62.9 | 79.0 |
| 86 | GCTCTAGTACAG-GTGTC**G**CTGG | C | T | 3.3 | 1,2 | 0.1 | 0.9 |
| 92 | **G**CTTCCGGGCCGCAGGGTCGCGGG | G | A | 14.1 | 15.4 | 6,8 | 21.5 |
| 97 | CCTCTAGACC-AGGGGTCGCA**G**G | G | A | 0 | 1.1 | 0 | 0.7 |
| 114 | GCTC-AGCCCTAGCTGTC**G**CTGG | C | T | 0 | 0.3 | 4.4 | 1.2 |
| 115 | GCTCT-G**G**GGGATGAGTCGCAGG | G | A | 44.5 | 51.7 | 29.4 | 34.2 |
| 123 | GTTCTTAGTCCAATGTGTCGCC**G**G | C | T | 0 | 0.3 | 2.9 | 0.8 |
| 124 | GCTTTTGATCGTAAGTGTC**G**CTGG | G | C | 4.6 | 5.3 | 20.7 | 12.2 |
| 126 | GGTCTCAGGCGCAGGTGTCG**C**GGG | G | C | 0 | 0 | 1.4 | 0.4 |
| 127 | GCTCTACAGCAGG**A**GGGTCGCGGG | T | C | 96.0 | 96.8 | 85.9 | 94.2 |
| 135 | GCC-TGGGCAGCATTGT**C**GCAGG | C | G | 44.9 | 45.5 | 57.9 | 45.7 |
| 142 | GCTCT-GTTCGTGCTG**T**CGCTGG | A | T | 5.6 | 18.6 | 44.1 | 23,9 |
| 142 | GCT**C**T-GTTCGTGCTGTCGCTGG | G | C | 5.6 | 18.3 | 43.9 | 23.9 |
| 142 | GCTCT-GTTCGTGCTGTC**G**CTGG | C | T | 0 | 0.9 | 12.5 | 3.4 |
| 146 | GCTCATAAGCAG**G**GCTGTCGCTGG | G | A | 10.6 | 17.4 | 40.9 | 27.9 |
| 147 | GATTCAGG**T**CCGCAGGGTCGCTGG | T | A | 0 | 0.3 | 2.8 | 0.8 |
| 150 | GCTCTTGGGAC**G**CCCAGTCGCTGG | G | A | 0.1 | 0 | 1.3 | 0.4 |
| 163 | GCA-GGGGCTGAGGC**G**TCGCAGG | C | T | 1.6 | 1.2 | 0.8 | 0.6 |
| 168 | GATCTGGGCAG—GTGTC**G**CTGG | G | A | 0 | 0 | 2.6 | 0.7 |
| 170 | GCTGCAGG**C**G—AGTGTCGCTGG | C | T | 21.0 | 28.9 | 9.5 | 31.5 |
| 173 | GCTGTTTGCC—AGTGTCGCT**G**G | G | A | 0 | 0.6 | 3.9 | 1.1 |
| 173 | GCTGTTTGCC—AGTGTC**G**CTGG | G | A | 1.8 | 0.1 | 3.1 | 0.8 |
| 179 | GCTG—GGCCCAAGGGT**C**GCAGG | G | A | 9.4 | 15.6 | 3.1 | 14.7 |
| 179 | GCTG—GGCCCAAGGGTCGC**A**GG | T | A | 0 | 2.9 | 0.1 | 0.6 |
| 181 | GCTC—GGCCTCGGTGTCG**C**GGG | G | A | 1.2 | 1.1 | 0.1 | 0.4 |
| 197 | GAACTGGGCA**A**CGAGGTGTCGCTGG | A | G | 38.4 | 28.1 | 15.1 | 31.8 |
| 201 | GCTCT—GCCAG**C**GAGTCGCTGG | C | T | 15.5 | 23.8 | 39.9 | 28.5 |
| 210 | GCTGTGGGCCCA—GGTC**G**CTGG | C | T | 0.1 | 2.4 | 23.2 | 6.5 |
| 218 | GC**T**CACAGCCGGGAGGTGTCGCCGG | A | G | 0.1 | 0.6 | 1.9 | 0.6 |
| 219 | G**C**TTCAGGGTGAAGTGAGTCGCAGG | G | A | 0.1 | 0.6 | 11.2 | 3.1 |
| 221 | G**A**TCCAGGCAGAGGGCTGTCGCTGG | A | G | 61.9 | 65.1 | 92.7 | 72.8 |
| 222 | GCTGAAAGCCG**G**CAGGTGTCGCAGG | G | A | 0.1 | 0.1 | 4.8 | 1.3 |
| 222 | GCTGAAAGCC**G**GCAGGTGTCGCAGG | G | A | 0 | 0 | 1.1 | 0.3 |
| 223 | GCTCCAGGCCCTG**C**CTTGTCGCTGG | C | A | 1.8 | 0.1 | 0.2 | 0.4 |
| 226 | CCTCTATTCCCC—TGTCGC**G**GG | G | C | 59.2 | 39.5 | 68.0 | 54.3 |
| 227 | GCTC**C**CGGGCCC—TGTCGCTGG | G | A | 0 | 0.6 | 5.9 | 1.6 |
| 228 | GTTTTTGTTC—AGT**G**TCGCGGG | C | T | 0 | 0 | 1.6 | 0.4 |
| 230 | CATCTGGGCT—TGTGT**C**GCCGG | C | T | 0 | 0 | 1.5 | 0.4 |
| 235 | GGTGCAGGCTGA—G**G**TCGCGGG | G | A | 0.2 | 1.2 | 5.2 | 4.6 |
| 239 | GCTT—C**C**CCAAATTGTCGCTGG | G | T | 1.5 | 7.1 | 24.4 | 8.7 |
| 242 | G—CTAGGCAGCCTGGTC**G**CCGG | G | T | 0 | 0 | 2.8 | 0.7 |
| 244 | GCTCT**C**GGCTCTCACCTGTCGCGGG | G | A | 43.0 | 54.9 | 43.0 | 49.9 |
| 248 | **G**CCATCTGTGGTGAAGCGTCGCCGG | C | T | 0.1 | 0.3 | 4.9 | 1.1 |
| 263 | AGTCTGGGGC—ATG**G**TCGCTGG | C | A | 0 | 1.7 | 10.9 | 3.1 |
| 265 | GTACCAGTTC—AGGGT**C**GCAGG | G | A | 33.8 | 24.8 | 11.3 | 23.8 |
| 265 | GT**A**CCAGTTC—AGGGTCGCAGG | T | A | 33.8 | 24.8 | 11.3 | 23.8 |
| 269 | CCTCTGGGGCTTCAGGGGTC**G**CAGG | C | T | 34.2 | 40.5 | 19.1 | 33.1 |
| 271 | GATCCAGGCTGCCAGAA**G**TCGCTGG | G | A | 0 | 0.1 | 1.2 | 0.3 |
