# Supplementary material for: In silico analysis of potential off-target sites to gene editing for Mucopolysaccharidosis type I using the CRISPR/Cas9 system: Implications for population-specific treatments
Source: PLoS One. 2022 Jan 24;17(1):e0262299. doi: 10.1371/journal.pone.0262299 (PMC8786118; doi:10.1371/journal.pone.0262299)
Supplement: S5 Table — Allele frequency is shown for the Brazilian population according to the ABraOM database. Position of the alternative allele in bold. The star signal (*) denotes variants not found in the 1000 Genomes database. (DOCX) [file pone.0262299.s006.docx]

**S5 Table**

| **ID** | **SEQUENCE** | **REF** | **ALT** | **BRAZIL** | **OBSERVATION** |
| --- | --- | --- | --- | --- | --- |
| 22 | GCCCTGGTC**C**GCTGTGTCGCTGG | G | T | 79,4 |  |
| 50 | GCCCTCGTCCGTC**T**TGTCGCAGG | T | C | 1,5 |  |
| 69 | GGA**C**CAGGCGGAGGCGTCGCGGG | C | T | 1,3 |  |
| 127 | GCTCTACAGCAGG**A**GGGTCGCGGG | T | C | 95,7 |  |
| 135 | GCC-TGGGCAGCATTGT**C**GCAGG | C | G | 46,6 |  |
| 143 | GCTGCAGGCGGG**A**GGGGTCGCGGG | A | G | 2,3 | * |
| 143 | GCTGCAGGCGGGAGGGG**T**CGCGGG | T | G | 7,2 | * |
| 163 | GCA-GGGG**C**TGAGGCGTCGCAGG | C | T | 1,4 | * |
| 170 | GCTGCAGG**C**G—AGTGTCGCTGG | C | T | 21,9 |  |
| 171 | GCTGCAGG**C**G—AGTGTCGCTGG | C | T | 1,4 | * |
| 181 | GCTC—GGCCTCGGTGTCG**C**GGG | G | A | 3,6 | * |
| 194 | **G**CTCTTCGGCCCAACGTGTCGCTGG | G | T | 1,2 | * |
| 201 | GCTCT—GCCAG**C**GAGTCGCTGG | C | T | 19,7 |  |
| 221 | G**A**TCCAGGCAGAGGGCTGTCGCTGG | A | G | 71,0 |  |
| 226 | CCTCTATTCCCC—TGTCGC**G**GG | G | C | 54,4 |  |
| 235 | GGTGCAGGCTGA—G**G**TCGCGGG | G | A | 1,7 |  |
| 244 | GCTCT**C**GGCTCTCACCTGTCGCGGG | G | A | 44,0 |  |
