# Supplementary material for: In silico analysis of potential off-target sites to gene editing for Mucopolysaccharidosis type I using the CRISPR/Cas9 system: Implications for population-specific treatments
Source: PLoS One. 2022 Jan 24;17(1):e0262299. doi: 10.1371/journal.pone.0262299 (PMC8786118; doi:10.1371/journal.pone.0262299)
Supplement: S6 Table — The table shows sequence identification tag (ID) following Table 1, the sequence with the alternative allele in bold, reference and alternative alleles, and the number of mismatches and indels without (no variant) or with the variant (variant). (DOCX) [file pone.0262299.s007.docx]

**S6 Table**

| **ID** | **SEQUENCE** | **REF** | **ALT** | **NO VARIANT** | **VARIANT** | **PAM** |
| --- | --- | --- | --- | --- | --- | --- |
| 5 | CCACTAGGCCAAAGT**GTA**GCTGG | TAC | T | 4/0 | 5/2 | - |
| 10 | CCACCAGGCTGCAGTGTC**G**CAGG | G | A | 5/0 | 6/0 | - |
| 11 | GGTCTGGGTGTAAGTGTC**G**CGGG | C | T | 5/0 | 6/0 | - |
| 11 | GGTCTGGGTGTAAGTGT**C**GCGGG | G | A | 5/0 | 6/0 | - |
| 12 | GCGCCCG**G**CCCGAGTGTCGCGGG | C | G | 5/0 | 6/0 | - |
| 16 | AATCCAGGTCGAAGGGTC**G**CCGG | C | T | 5/0 | 6/0 | - |
| 22 | GCCCTGGTC**C**GCTGTGTCGCTGG | G | T | 5/0 | 6/0 | - |
| 30 | GCTGAAGGCCTGAGGGTCGC**C**GG | C | T | 5/0 | 5/0 | TGG |
| 34 | GATCTAGGCTAAGGAGTC**G**CAGG | C | T | 5/0 | 6/0 | - |
| 37 | **GCTCCT**CCAGGAAGTGTCGCAGG | GCTCCT | G | 6/0 | 5/5 | - |
| 37 | GCTCCTC**CAGGA**AGTGTCGCAGG | CAGGA | C | 6/0 | 4/4 | - |
| 40 | TGTCCTTGCTGAAGTGTC**G**CAGG | G | C | 6/0 | 7/0 | - |
| 44 | AATCCAGG**T**TGAAGGGTCGCTGG | T | C | 6/0 | 5/0 | - |
| 45 | GCCCTA**GGCA**TATCAGTCGCTGG | GGCA | G | 6/0 | 5/3 | - |
| 48 | CCTCGTCGCCGCTGTGTCG**C**TGG | G | C | 6/0 | 7/0 | - |
| 48 | CCTC**G**TCGCCGCTGTGTCGCTGG | C | G | 6/0 | 6/0 | - |
| 50 | GCCCTCGTCCGTC**T**TGTCGCAGG | T | C | 6/0 | 6/0 | - |
| 57 | ACTCTGTGCTCATGTGTC**G**CAGG | C | T | 6/0 | 7/0 | - |
| 58 | ATCCTAGGCCCCTGTGTC**G**CAGG | C | T | 6/0 | 7/0 | - |
| 61 | AATCCAGGTTGAAGAGTC**G**CTGG | G | A | 6/0 | 7/0 | - |
| 69 | GGA**C**CAGGCGGAGGCGTCGCGGG | C | T | 6/0 | 7/0 | - |
| 70 | AATCCAGGTCAAA**G**GGTCGCTGG | G | A | 6/0 | 7/0 |  |
| 71 | AATCCAGGTTGAAGGGTC**G**CTGG | C | T | 6/0 | 7/0 | - |
| 73 | T**T**TCTAGCCAGGACTGTCGCTGG | A | G | 6/0 | 5/0 | - |
| 75 | GCTC-AGGCTGAAGGGT**C**GCAGG | C | T | 2/1 | 3/1 | - |
| 75 | GCTC-AGGCTGAA**G**GGTCGCAGG | G | C | 2/1 | 3/1 | - |
| 82 | CCCCTAGGCCTAAG-GTCG**C**GGG | C | T | 3/1 | 4/1 | - |
| 86 | GCTCTAGTACAG-GTGTC**G**CTGG | C | T | 4/1 | 5/1 | - |
| 92 | **G**CTTCCGGGCCGCAGGGTCGCGGG | G | A | 4/1 | 5/1 | - |
| 114 | GCTC-AGCCCTAGCTGTC**G**CTGG | C | T | 4/1 | 4/1 | - |
| 115 | GCTCT-G**G**GGGATGAGTCGCAGG | G | A | 4/1 | 5/1 | - |
| 124 | GCTTTTGATCGTAAGTGTC**G**CTGG | G | C | 4/1 | 5/1 | - |
| 126 | GGTCTCAGGCGCAGGTGTCG**C**GGG | G | C | 4/1 | 5/1 | - |
| 127 | GCTCTACAGCAGG**A**GGGTCGCGGG | T | C | 4/1 | 5/1 | - |
| 135 | GCC-TGGGCAGCATTGT**C**GCAGG | C | G | 5/1 | 6/1 | - |
| 142 | GCTCT-GTTCGTGCTG**T**CGCTGG | A | T | 5/1 | 6/1 | - |
| 142 | GCT**C**T-GTTCGTGCTGTCGCTGG | G | C | 5/1 | 6/1 | - |
| 142 | GCTCT-GTTCGTGCTGTC**G**CTGG | C | T | 5/1 | 6/1 | - |
| 146 | GCTCATAAGCAG**G**GCTGTCGCTGG | G | A | 5/1 | 4/1 | - |
| 147 | GATTCAGG**T**CCGCAGGGTCGCTGG | T | A | 5/1 | 5/1 | - |
| 150 | GCTCTTGGGAC**G**CCCAGTCGCTGG | G | A | 6/1 | 7/1 | - |
| 163 | GCA-GGGGCTGAGGC**G**TCGCAGG | C | T | 6/1 | 7/1 | - |
| 168 | GATCTGGGCAG—GTGTC**G**CTGG | G | A | 3/2 | 4/2 | - |
| 170 | GCTGCAGG**C**G—AGTGTCGCTGG | C | T | 3/2 | 4/2 | - |
| 173 | GCTGTTTGCC—AGTGTC**G**CTGG | G | A | 3/2 | 4/2 | - |
| 179 | GCTG—GGCCCAAGGGT**C**GCAGG | G | A | 3/2 | 4/2 | - |
| 179 | GCTG—GGCCCAAGGGTCGC**A**GG | T | A | 3/2 | 3/2 | TGG |
| 181 | GCTC—GGCCTCGGTGTCG**C**GGG | G | A | 3/2 | 4/2 | - |
| 197 | GAACTGGGCA**A**CGAGGTGTCGCTGG | A | G | 4/2 | 4/2 | - |
| 201 | GCTCT—GCCAG**C**GAGTCGCTGG | C | T | 4/2 | 4/2 | - |
| 210 | GCTGTGGGCCCA—GGTC**G**CTGG | C | T | 4/2 | 5/2 | - |
| 218 | GC**T**CACAGCCGGGAGGTGTCGCCGG | A | G | 4/2 | 5/2 | - |
| 219 | G**C**TTCAGGGTGAAGTGAGTCGCAGG | G | A | 4/2 | 5/2 | - |
| 221 | G**A**TCCAGGCAGAGGGCTGTCGCTGG | A | G | 4/2 | 4/2 | - |
| 222 | GCTGAAAGCCG**G**CAGGTGTCGCAGG | G | A | 4/2 | 4/2 | - |
| 222 | GCTGAAAGCC**G**GCAGGTGTCGCAGG | G | A | 4/2 | 5/2 | - |
| 223 | GCTCCAGGCCCTG**C**CTTGTCGCTGG | C | A | 4/2 | 3/2 | - |
| 226 | CCTCTATTCCCC—TGTCGC**G**GG | G | C | 5/2 | 5/2 | CGG |
| 227 | GCTC**C**CGGGCCC—TGTCGCTGG | G | A | 5/2 | 4/2 | - |
| 228 | GTTTTTGTTC—AGT**G**TCGCGGG | C | T | 5/2 | 6/2 | - |
| 230 | CATCTGGGCT—TGTGT**C**GCCGG | C | T | 5/2 | 6/2 | - |
| 235 | GGTGCAGGCTGA—G**G**TCGCGGG | G | A | 5/2 | 6/2 | - |
| 239 | GCTT—C**C**CCAAATTGTCGCTGG | G | T | 5/2 | 5/2 | - |
| 242 | G—CTAGGCAGCCTGGTC**G**CCGG | G | T | 5/2 | 6/2 | - |
| 244 | GCTCT**C**GGCTCTCACCTGTCGCGGG | G | A | 5/2 | 5/2 |  |
| 248 | **G**CCATCTGTGGTGAAGCGTCGCCGG | C | T | 5/2 | 6/2 | - |
| 263 | AGTCTGGGGC—ATG**G**TCGCTGG | C | A | 6/2 | 7/2 | - |
| 265 | GTACCAGTTC—AGGGT**C**GCAGG | G | A | 6/2 | 7/2 | - |
| 265 | GT**A**CCAGTTC—AGGGTCGCAGG | T | A | 6/2 | 5/2 | - |
| 269 | CCTCTGGGGCTTCAGGGGTC**G**CAGG | C | T | 6/2 | 7/2 | - |
| 271 | GATCCAGGCTGCCAGAA**G**TCGCTGG | G | A | 6/2 | 7/2 | - |
| 273 | GCTTATGCCAGTAGTGTCGCAG**T** | T | G | 6/0 | 6/0 | AGG |
